# Supplementary material for: Ternary aromatic and anti-aromatic clusters derived from the hypho species [Sn2Sb5]3−
Source: Nat Commun. 2021 Jul 22;12:4465. doi: 10.1038/s41467-021-24706-4 (PMC8298489; doi:10.1038/s41467-021-24706-4)
Supplement: Supplementary file 1 — Supplementary Information [file 41467_2021_24706_MOESM1_ESM.pdf]

Ternary Aromatic and Anti-aromatic Clusters Derived from the *Hypho* Species $[Sn_2Sb_5]^{3-}$

Yu-He Xu, Nikolay V. Tkachenko, Ivan A. Popov, Lei Qiao, Alvaro Muñoz-Castro, Alexander I. Boldyrev, and Zhong-Ming Sun

Content

Table of Contents S1
1. Crystallographic Supplementary Information S2
2. ESI-MS Studies S10
3. Energy Dispersive X-ray (EDX) Spectroscopic Analysis S18
4. Supplementary Computational Data S22

1. Crystallographic Supplementary Information

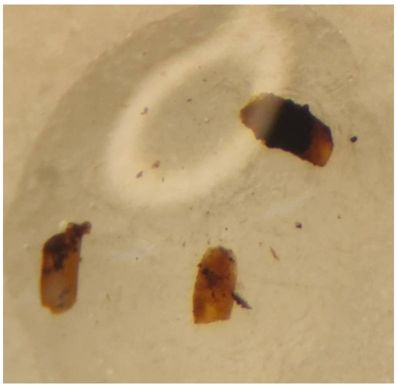

(1)

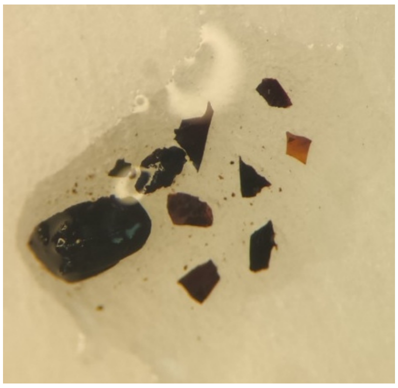

(2)

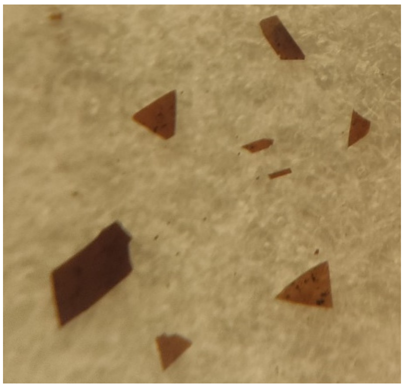

(3)

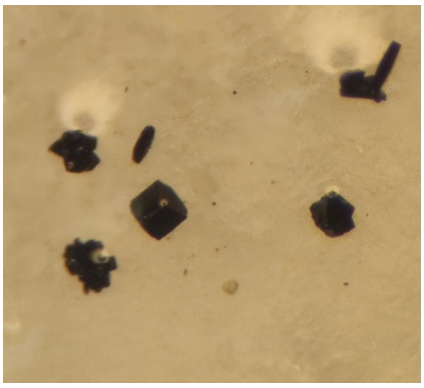

(4)

Supplementary Figure 1. Crystals of (1) $[K(2.2.2\text{-crypt})]_3[Cr_2(CO)_6Sn_2Sb_5]$, (2) $[K(2.2.2\text{-crypt})]_3[Mo_2(CO)_6Sn_2Sb_5]$, (3) $[K(2.2.2\text{-crypt})]_4[(AgSn_2Sb_5)_2]$ and (4) $[K(2.2.2\text{-crypt})]_4[(CuSn_2Sb_5)_2]$, dispersed in silicon oil, respectively.

Supplementary Table 1. X-ray measurements and structure solutions of $[K(2.2.2\text{-crypt})]_3[Cr_2(CO)_6Sn_2Sb_5]$ (**1'**), $[K(2.2.2\text{-crypt})]_3[Mo_2(CO)_6Sn_2Sb_5]$ (**2'**), $[K(2.2.2\text{-crypt})]_4[(AgSn_2Sb_5)_2]$ (**3'**) and $[K(2.2.2\text{-crypt})]_4[(CuSn_2Sb_5)_2]$ (**4'**).

Compound	1'	2'	3'	4'
Empirical formula	$C_{60}H_{107}N_6O_{24}K_3Cr_2Sn_2Sb_5$	$C_{60}H_{108}N_6O_{24}K_3Mo_2Sn_2Sb_5$	$C_{36}H_{70.1}N_4O_{12}K_2AgSn_2Sb_5$	$C_{36}H_{63}N_4O_{12}K_2CuSn_2Sb_5$
Formula weight	2363.94	2452.83	1783.17	1731.77
Crystal system	Monoclinic	Monoclinic	Triclinic	Monoclinic
Space group	$P2_1$/n	$P2_1$/n	P-1	$P2_1$/n
a /Å	13.4845(2)	13.58390(10)	15.3848(3)	16.2372(2)
b /Å	51.0677(7)	51.2152(4)	15.7304(3)	23.2882(2)
c /Å	14.8974(2)	14.94470(10)	16.8958(3)	16.7019(2)
α/°	90	90	118.449(2)	90

β/°	98.3610(10)	99.0880(10)	95.915(2)	115.8931(15)
γ/°	90	90	111.955(2)	90
V /Å3	10149.6(3)	10266.54(13)	2944.72(12)	5681.56(13)
Z	4	4	2	4
ρcalc /g·cm-3	1.547	1.587	2.011	2.025
μ(CuKα) /mm-1	17.510	17.568	28.819	27.569
F(000)	4644.0	4792.0	1700.0	3300.0
2Θ range /°	7.476 to 133.2	7.92 to 151.984	6.640 to 146.370	7.386 to 133.998
Reflections collected / unique	39438/14449	55943/32989	30779/17061	27626/18783
Data / restraints / parameters	17575/2657/913	20704/7/919	11486/188/657	10104/2478/703
R1/wR2 (I>2σ(I))a	0.0771; 0.2152	0.0656; 0.1714	0.0716; 0.1998	0.0606;0.1686
R1/wR2 (all data)	0.0902; 0.2267	0.0703; 0.1739	0.0798; 0.2076	0.0681;0.1764
GooF (all data)b	1.021	1.048	1.023	1.032
Data completeness	0.981	0.967	0.966	0.998
Max. peak/hole /e-·Å-3	3.75/-1.48	1.66/-1.65	2.90/-1.34	1.98/-1.36
CCDC	2080455	2080457	2080458	2080459

[a] $R_1 = = \sum||F_o|-|F_c||/\sum|F_o|$; $wR_2 = \{\sum w[(F_o)^2-(F_c)^2]^2/\sum w[(F_o)^2]^2\}^{1/2}$

[b] $GooF = \{\sum w[(Fo)^2-(Fc)^2]^2/(n-p)\}^{1/2}$

Supplementary Table 2. Selected bond lengths (in Å) of the experimental and optimized at the PBE0/def2-TZVP level of theory structures of $[Cr_2(CO)_6Sn_2Sb_5]^{3-}$.

	Experimental	C_{2v}-$[Cr_2(CO)_6Sn_2Sb_5]^{3-}$
	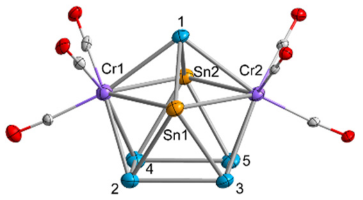	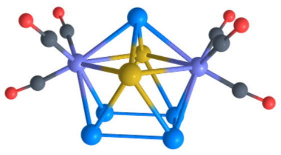

Sb1-Sn1	2.7914(8)	*2.7792*
Sb1-Sn2	2.7806(8)	*2.7792*
Sn1-Sb2	2.9230(9)	*2.9365*
Sn1-Sb3	2.9330(9)	*2.9365*
Sn2-Sb4	2.9203(8)	*2.9365*
Sn2-Sb5	2.9117(9)	*2.9365*
Sb2-Sb3	2.9468(10)	*2.9434*
Sb4-Sb5	2.9375(9)	*2.9434*
Sb2-Sb4	2.7851(9)	*2.8179*
Sb3-Sb5	2.7873(10)	*2.8179*
Cr1-Sb1	2.8513(12)	*2.9091*
Cr2-Sb1	2.8809(12)	*2.9091*
Cr1-Sn1	3.1268(13)	*3.1837*
Cr1-Sn2	3.2035(12)	*3.1837*
Cr2-Sn1	3.1590(12)	*3.1837*
Cr2-Sn2	3.1268(13)	*3.1837*
Cr1-Sb2	2.8364(12)	*2.8539*
Cr1-Sb4	2.8539(12)	*2.8539*
Cr2-Sb3	2.8532(12)	*2.8539*
Cr2-Sb5	2.8487(12)	*2.8539*

Supplementary Table 3. Selected bond lengths (in Å) of the experimental and optimized at the PBE0/def2-TZVP level of theory structures of $[Mo_2(CO)_6Sn_2Sb_5]^{3-}$.

	Experimental<br>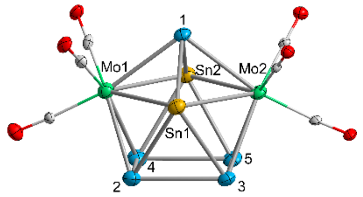	C_{2v}-$[Mo_2(CO)_6Sn_2Sb_5]^{3-}$<br>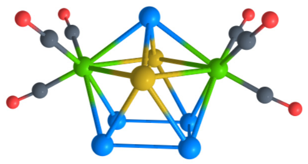
Sb1-Sn1	2.8131(7)	*2.8129*
Sb1-Sn2	2.8110(7)	*2.8129*
Sn1-Sb2	2.9560(7)	*2.9789*
Sn1-Sb3	2.9686(7)	*2.9789*
Sn2-Sb4	2.9513(7)	*2.9789*
Sn2-Sb5	2.9504(8)	*2.9789*
Sb2-Sb3	2.9243(8)	*2.9290*
Sb4-Sb5	2.9164(8)	*2.9290*
Sb2-Sb4	2.8057(8)	*2.8330*
Sb3-Sb5	2.8008(9)	*2.8330*
Mo1-Sb1	2.9437(7)	*3.0136*
Mo2-Sb1	2.9536(8)	*3.0136*
Mo1-Sn1	3.1895(8)	*3.2590*
Mo1-Sn2	3.2395(8)	*3.2590*
Mo2-Sn1	3.1912(8)	*3.2590*
Mo2-Sn2	3.1775(8)	*3.2590*
Mo1-Sb2	2.9260(8)	*2.9711*

Mo1-Sb4	2.9368(8)	*2.9711*
Mo2-Sb3	2.9374(8)	*2.9711*
Mo2-Sb5	2.9360(8)	*2.9711*

Supplementary Table 4. Selected bond lengths (in Å) of the experimental and optimized at the PBE0/def2-TZVP level of theory structures of $[(AgSn_2Sb_5)_2]^{4-}$.

	Experimental	C_{2h}-$[(AgSn_2Sb_5)_2]^{4-}$
	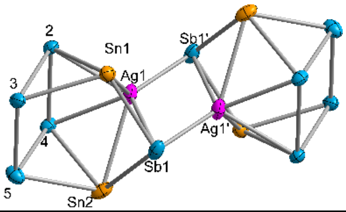	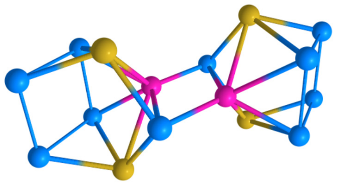
Sb1-Sn1	2.835(4)	*2.8623*
Sb1-Sn2	2.790(4)	*2.8623*
Sn1-Sb2	2.945(4)	*2.9578*
Sn2-Sb4	2.898(4)	*2.9578*
Sn1-Sb3	2.846(4)	*2.8938*
Sn2-Sb5	2.860(4)	*2.8938*
Sb2-Sb3	2.878(2)	*2.8628*
Sb4-Sb5	2.894(2)	*2.8628*
Sb2-Sb4	2.930(3)	*2.9321*
Sb3-Sb5	2.790(3)	*2.8845*
Ag1-Ag1'	2.802(2)	*2.8510*
Ag1-Sb1	2.9169(15)	*2.9242*
Ag1-Sn1	3.025(4)	*3.1711*
Ag1-Sn2	2.970(3)	*3.1711*
Ag1-Sb2	2.875(3)	*2.9681*
Ag1-Sb4	2.874(2)	*2.9681*
Ag1'-Sb1	2.7581(14)	*2.8500*

Supplementary Table 5. Selected bond lengths (in Å) of the experimental and optimized at the PBE0/def2-TZVP level of theory structures of $[(CuSn_2Sb_5)_2]^{4-}$.

	Experimental	C_{2h}-$[(CuSn_2Sb_5)_2]^{4-}$
	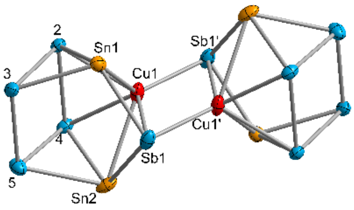	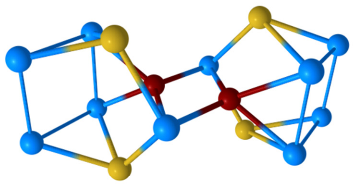

Sb1-Sn1	2.8190(10)	*2.8551*
Sb1-Sn2	2.8252(13)	*2.8551*
Sn1-Sb2	2.9124(14)	*2.9274*
Sn2-Sb4	2.9097(14)	*2.9274*
Sn1-Sb3	2.8373(10)	*2.9023*
Sn2-Sb5	2.8423(10)	*2.9023*
Sb2-Sb3	2.9181(9)	*2.8771*
Sb4-Sb5	2.9226(9)	*2.8771*
Sb2-Sb4	2.9437(9)	*2.9251*
Sb3-Sb5	2.8153(9)	*2.8775*
Cu1-Cu1’	2.547	*2.6150*
Cu1-Sb1	2.6707(17)	*2.6866*
Cu1-Sn1	2.8236(17)	*2.9780*
Cu1-Sn2	2.8522(17)	*2.9780*
Cu1-Sb2	2.6832(16)	*2.7496*
Cu1-Sb4	2.6866(18)	*2.7496*
Cu1-Sb1’	2.620	*2.7040*

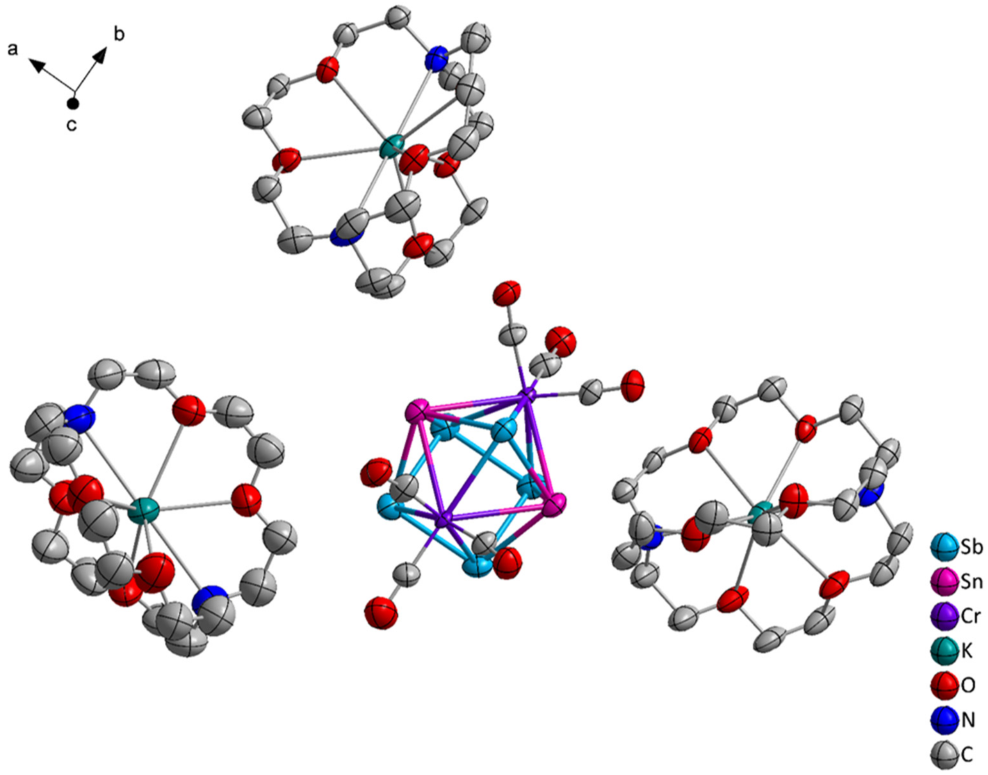

Supplementary Figure 2. Asymmetric unit of compound **1’**. Thermal ellipsoids are set at 50%.

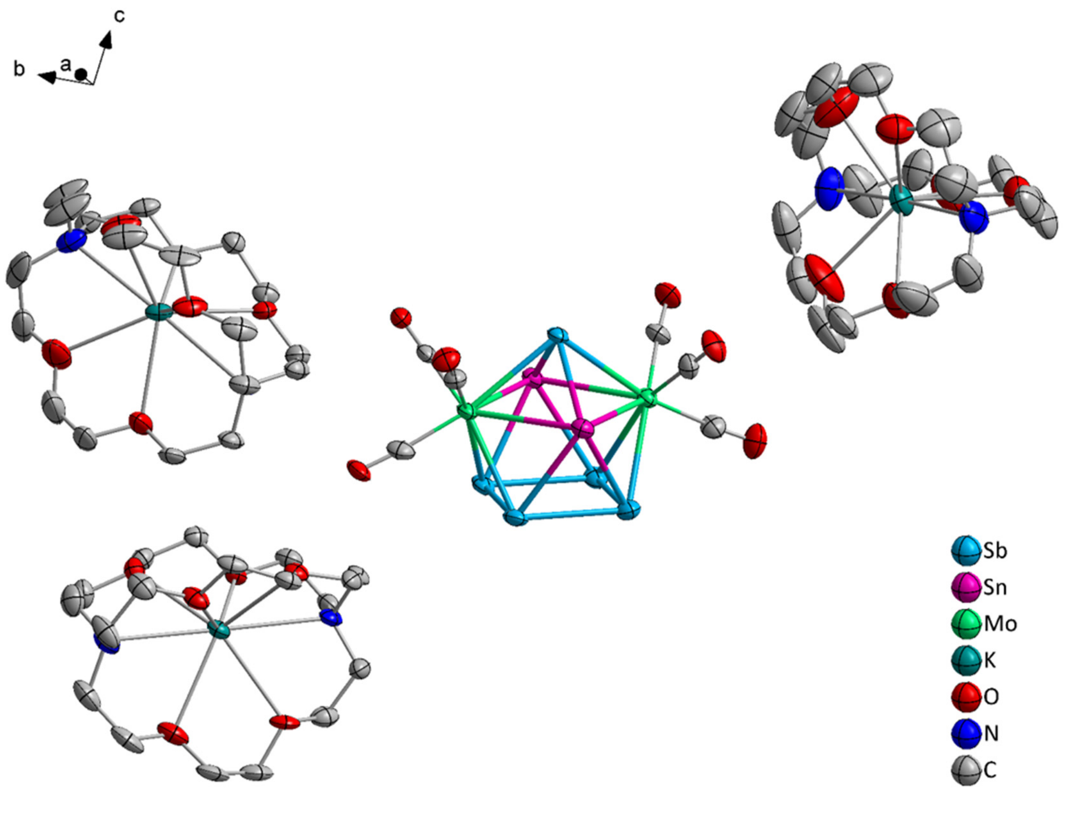

Supplementary Figure 3. Asymmetric unit of compound **2’**. Thermal ellipsoids are set at 50%.

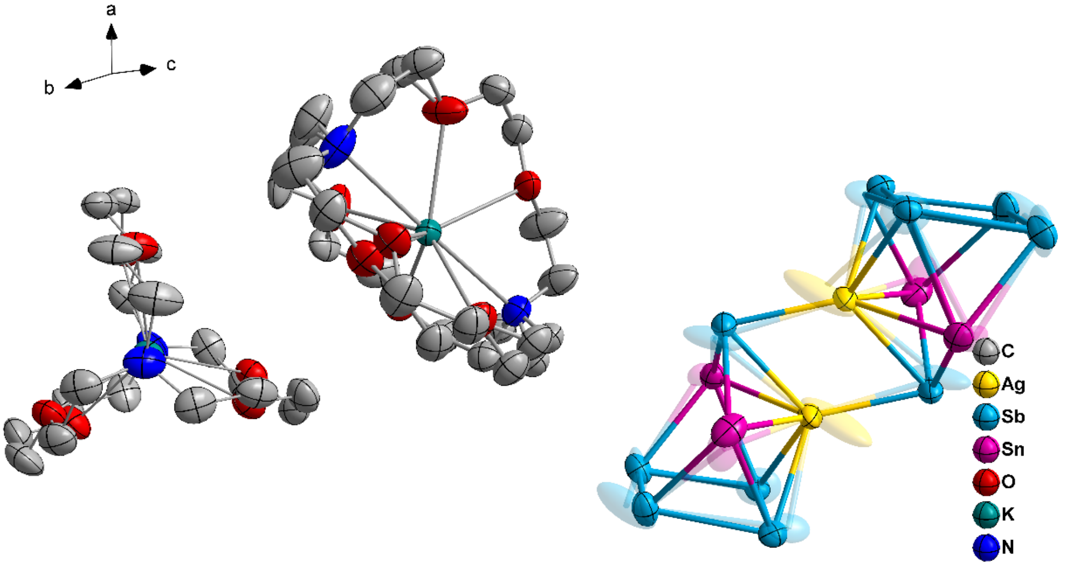

Supplementary Figure 4. Asymmetric unit of compound **3’**. Thermal ellipsoids are set at 50% and the minor components are omitted for clarity. The cluster **3** is slightly disordered and contains two types of $[(AgSn_2Sb_5)_2]^{4-}$. The solid ellipsoids represent the occupation of 83%, while the transparent ones take the remaining share.

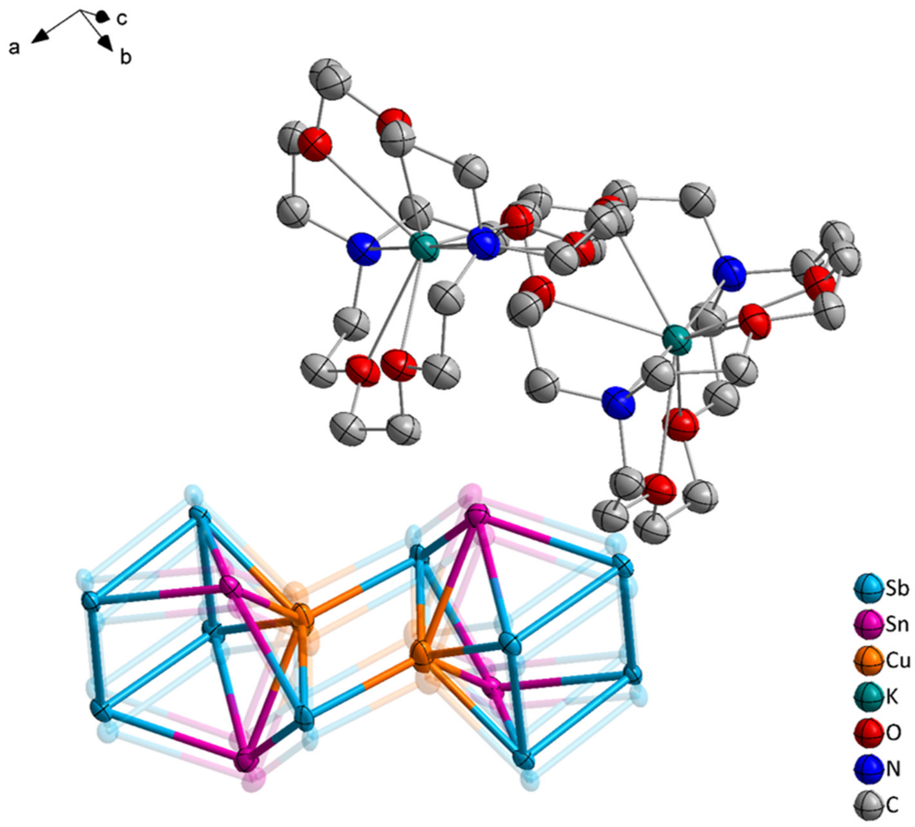

Supplementary Figure 5. Asymmetric unit of compound **4'**. Thermal ellipsoids are set at 50% and the minor components are omitted for clarity. The cluster **4** is slightly disordered and contains three types of $[(CuSn_2Sb_5)_2]^{4-}$. The solid ellipsoids represent the occupation of 88%, while the two transparent types each account for 6%.

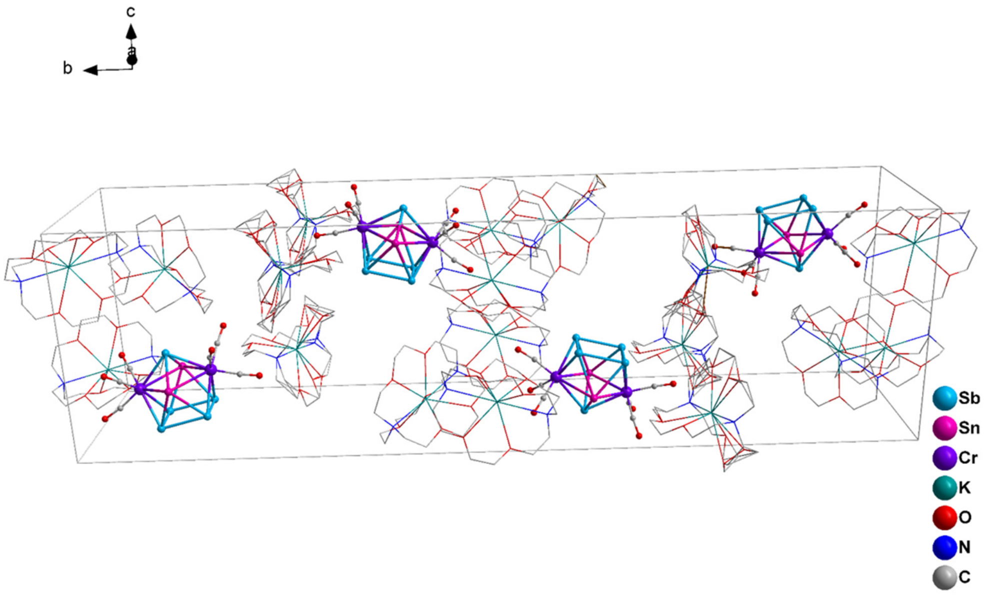

Supplementary Figure 6. Unit cell of compound **1'**.

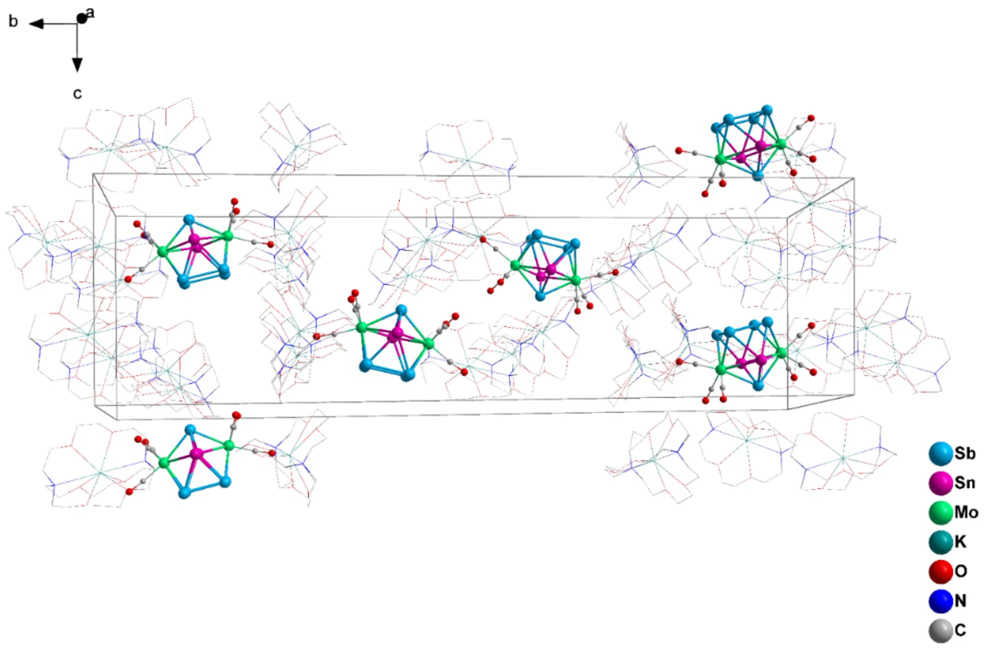

Supplementary Figure 7. Unit cell of compound **2’**.

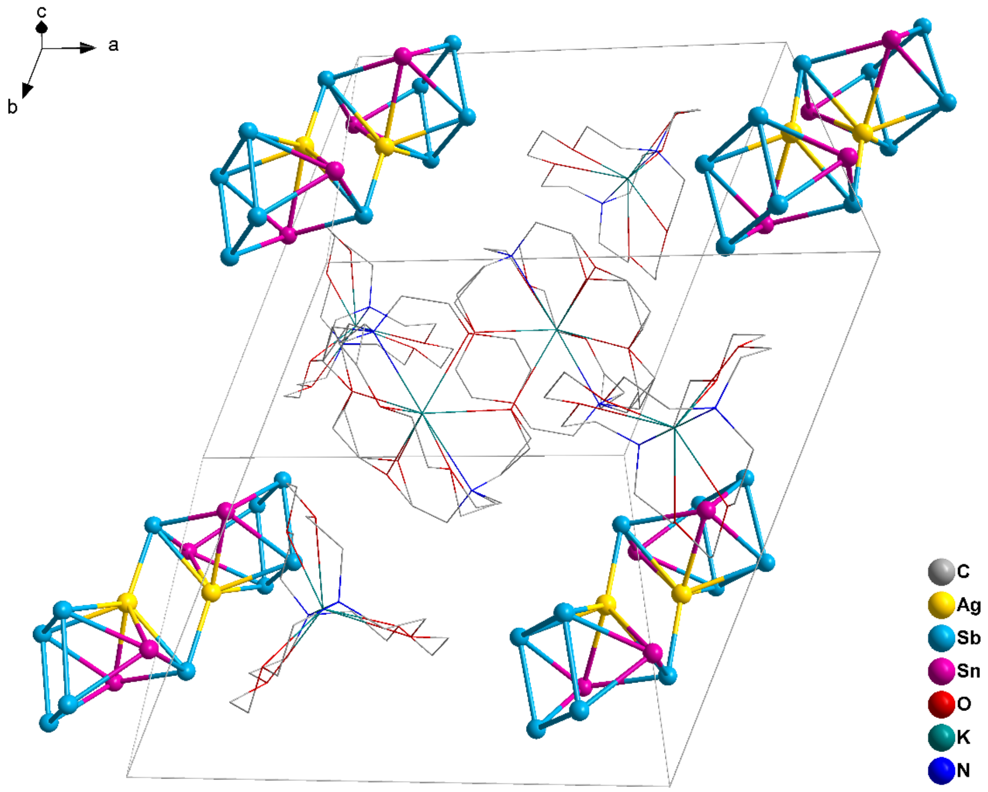

Supplementary Figure 8. Unit cell of compound **3’**. Minor component in the cluster site are omitted for clarity.

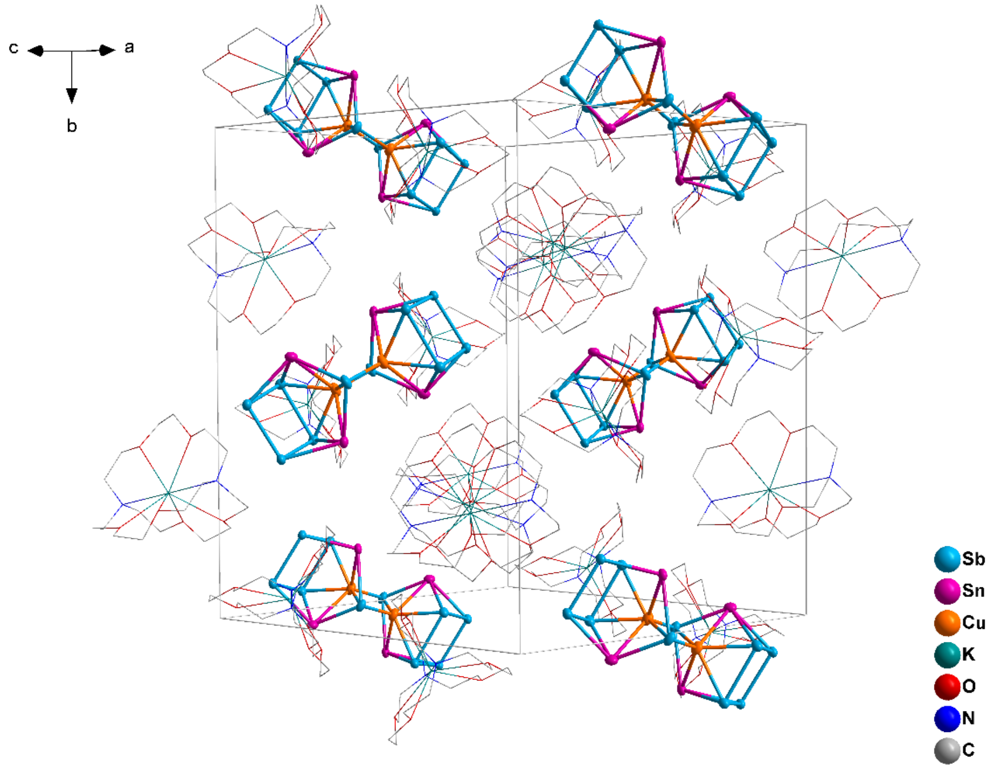

Supplementary Figure 9. Unit cell of compound **4’**. Minor component in the cluster site are omitted for clarity.

2. ESI-MS Studies

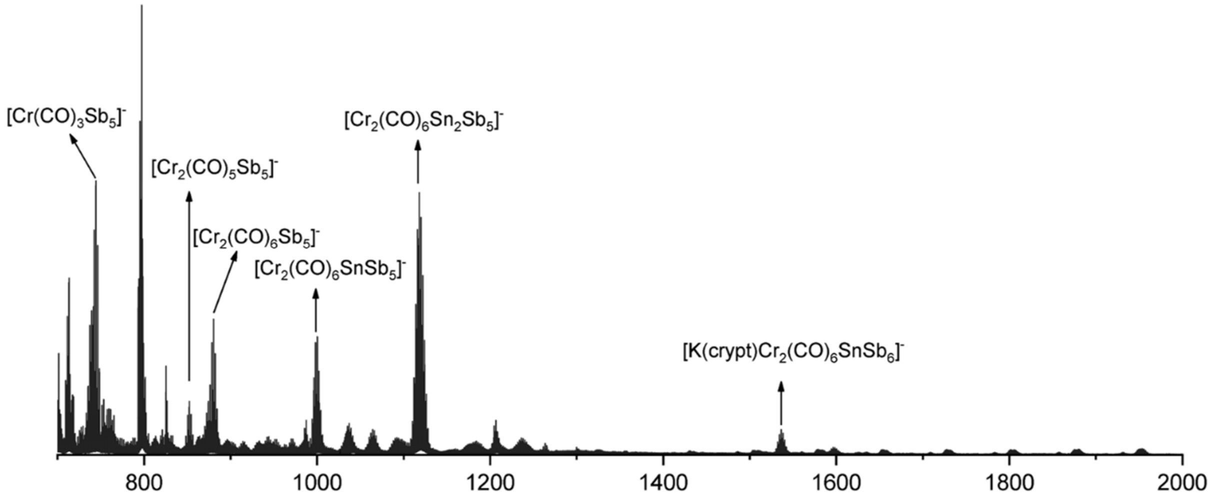

Supplementary Figure 10. Overview ESI (-) mass spectrum for compound **1’**. The sample was obtained from a freshly dissolved crystalline compound **1’** in DMF.

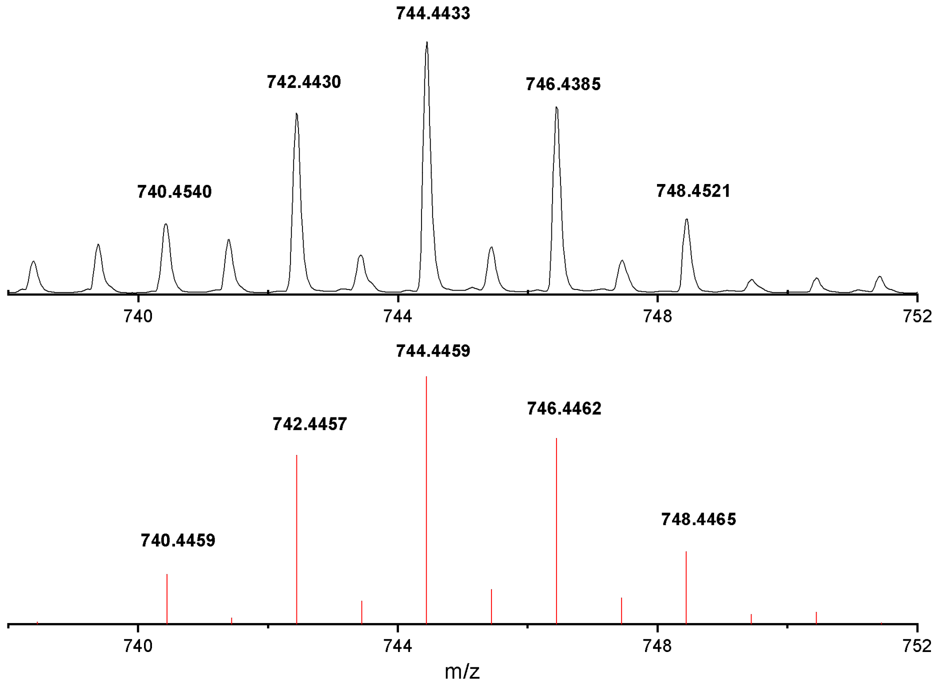

Supplementary Figure 11. Measured (top) and simulated (bottom) spectrum of the fragment $[Cr(CO)_3Sb_5]^-$.

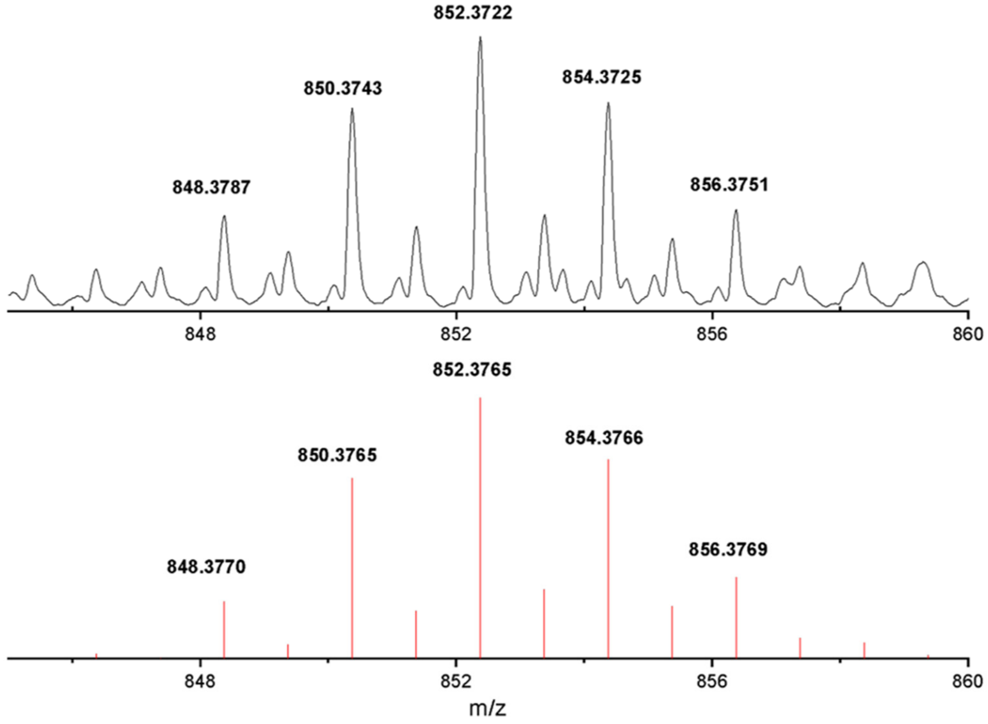

Supplementary Figure 12. Measured (top) and simulated (bottom) spectrum of the fragment $[Cr_2(CO)_5Sb_5]^-$.

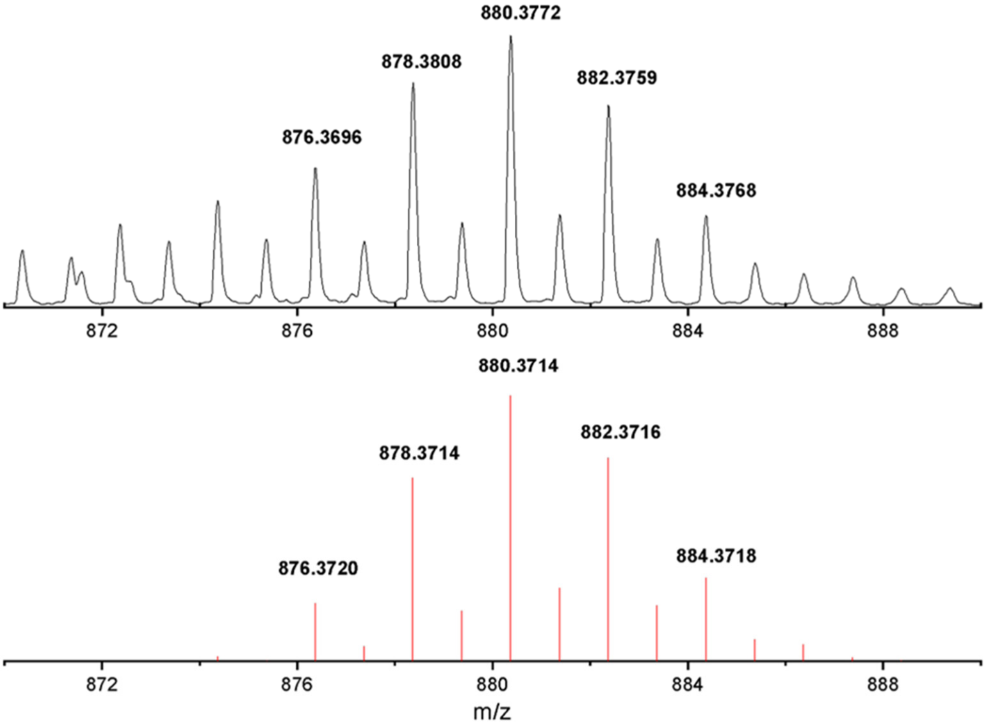

Supplementary Figure 13. Measured (top) and simulated (bottom) spectrum of the fragment $[Cr_2(CO)_6Sb_5]^-$.

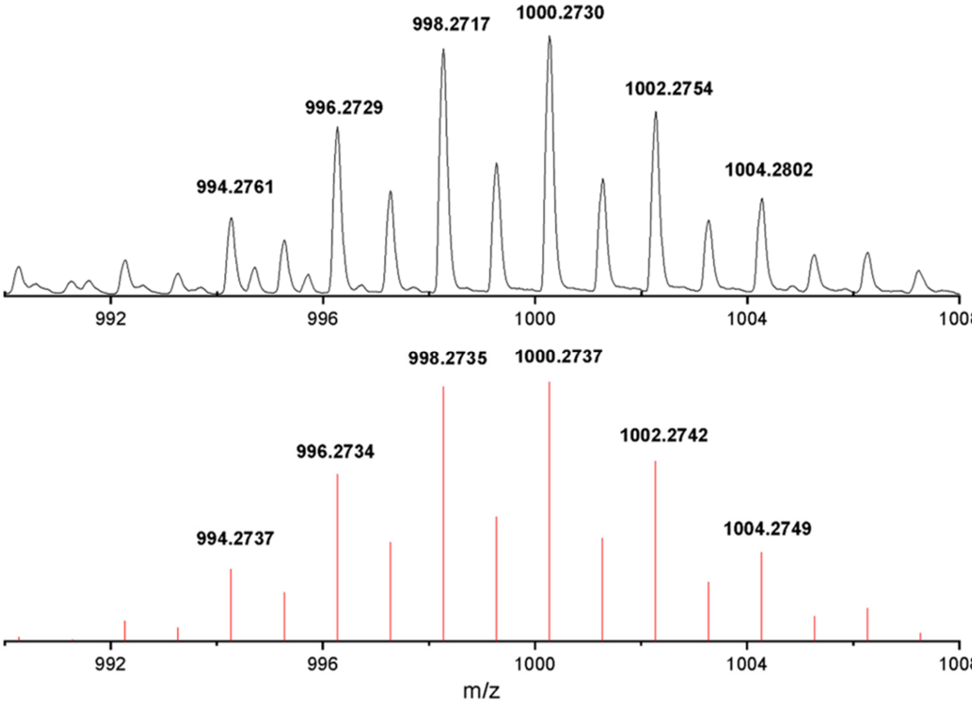

Supplementary Figure 14. Measured (top) and simulated (bottom) spectrum of the fragment $[Cr_2(CO)_6SnSb_5]^-$.

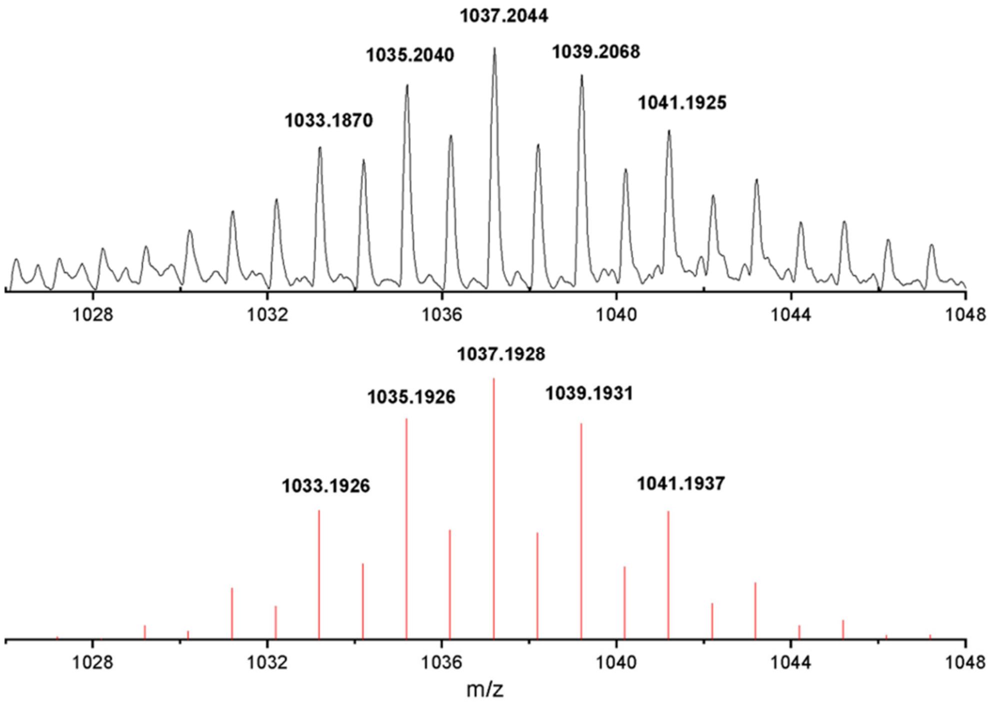

Supplementary Figure 15. Measured (top) and simulated (bottom) spectrum of the fragment $[Cr_2(CO)_3SnSb_6]^-$.

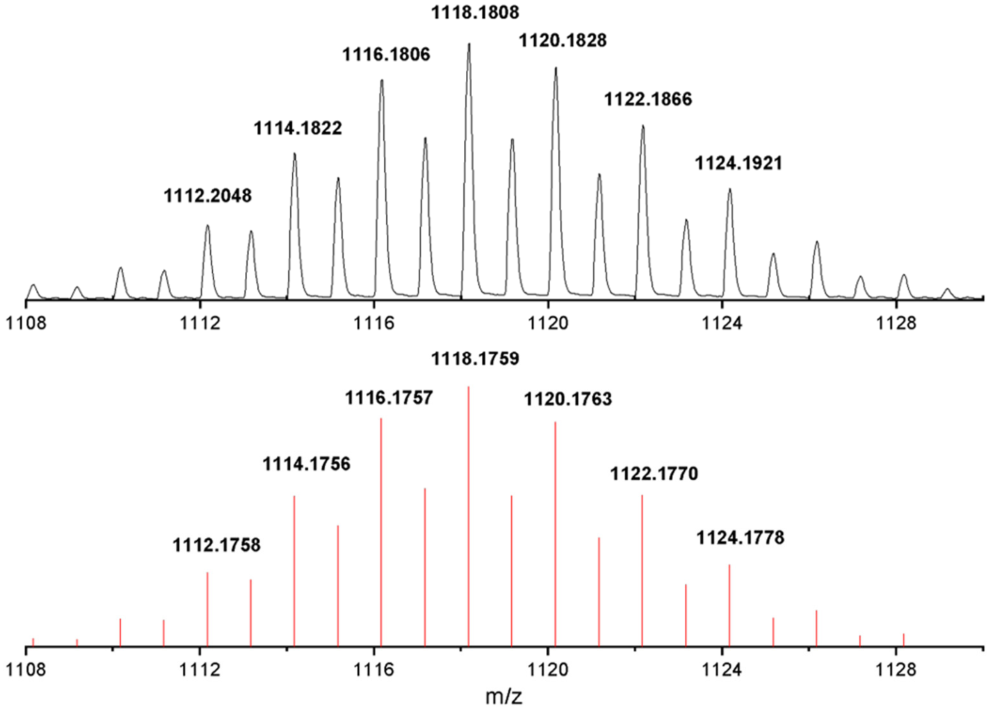

Supplementary Figure 16. Measured (top) and simulated (bottom) spectrum of the fragment $[Cr_2(CO)_6Sn_2Sb_5]^-$.

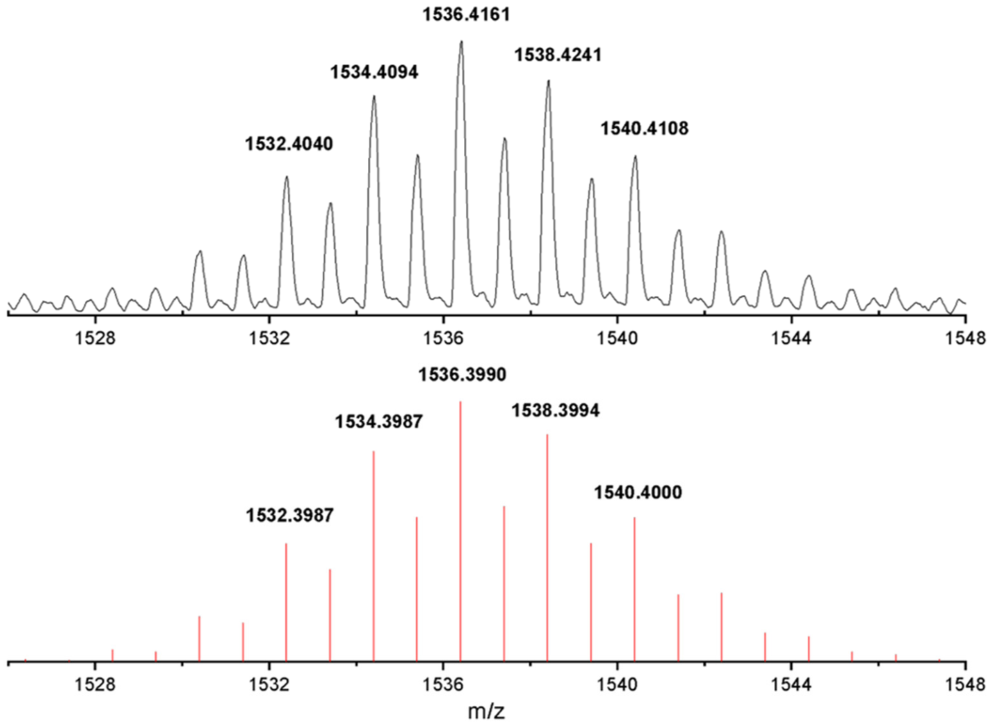

Supplementary Figure 17. Measured (top) and simulated (bottom) spectrum of the fragment $[K(crypt)Cr_2(CO)_6SnSb_6]^-$.

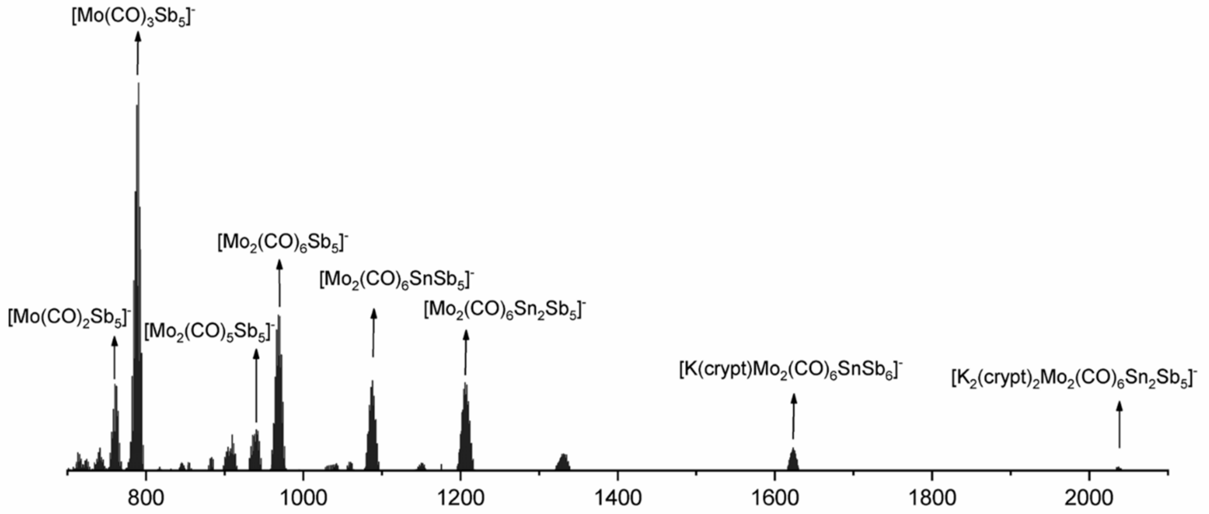

Supplementary Figure 18. Overview ESI (-) mass spectrum for compound **2’**. The sample was obtained from a freshly dissolved crystalline compound **2’** in DMF.

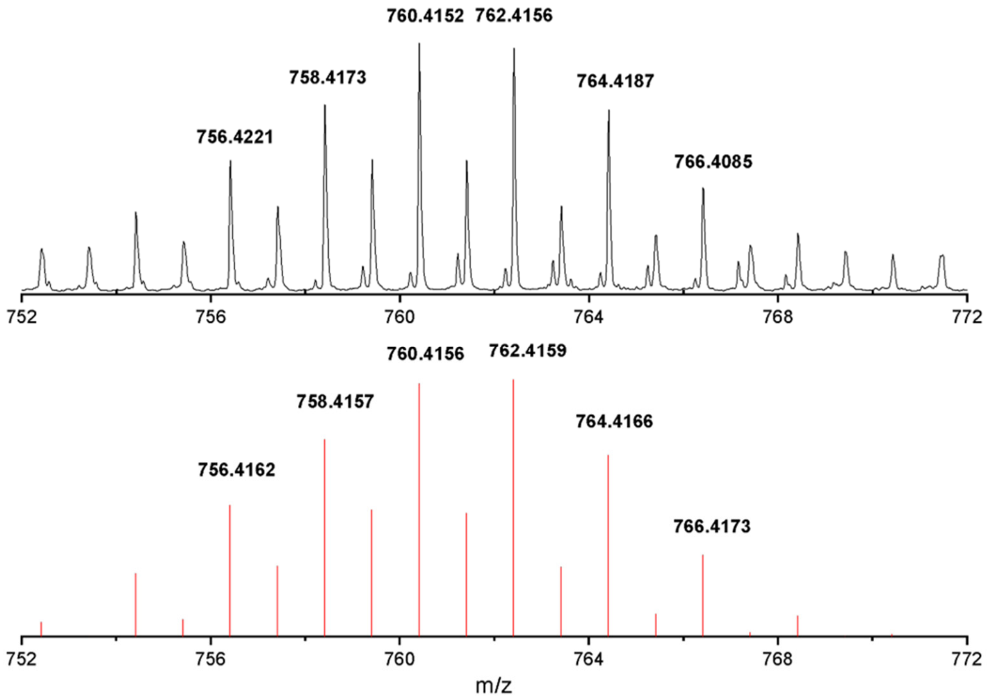

Supplementary Figure 19. Measured (top) and simulated (bottom) spectrum of the fragment $[Mo(CO)_2Sb_5]^-$.

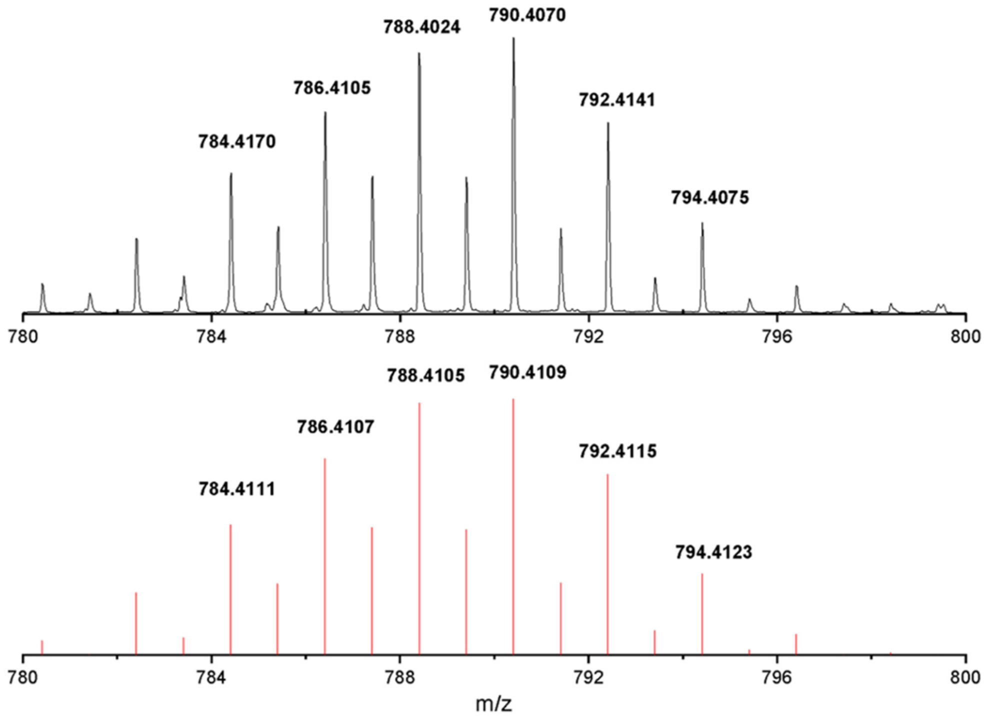

Supplementary Figure 20. Measured (top) and simulated (bottom) spectrum of the fragment $[Mo(CO)_3Sb_5]^-$.

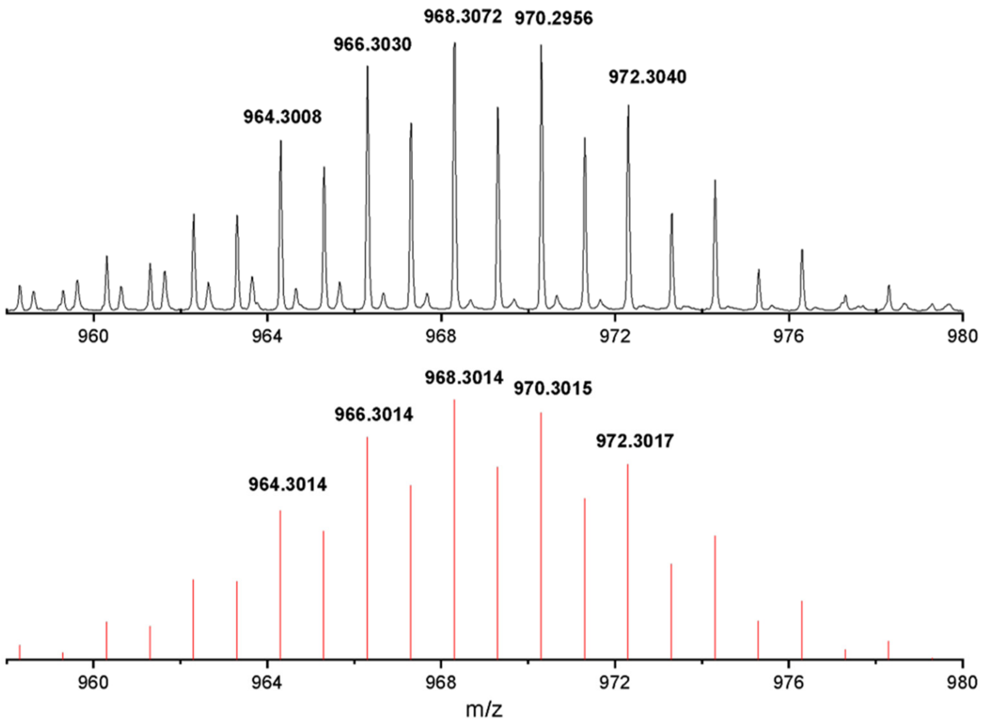

Supplementary Figure 21. Measured (top) and simulated (bottom) spectrum of the fragment $[Mo_2(CO)_6Sb_5]^-$.

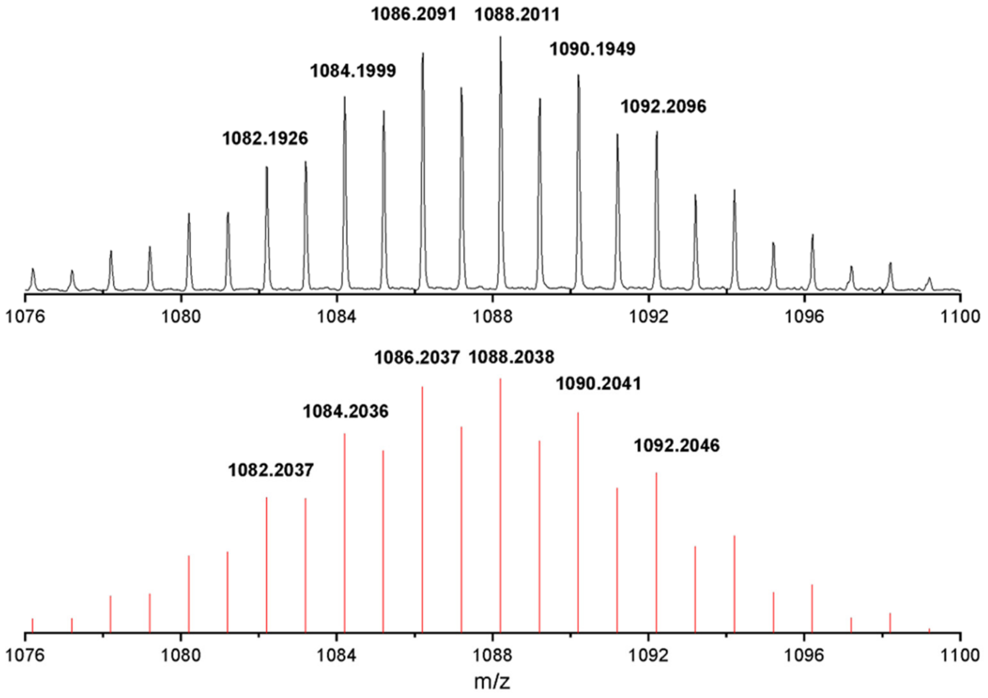

Supplementary Figure 22. Measured (top) and simulated (bottom) spectrum of the fragment $[Mo_2(CO)_6SnSb_5]^-$.

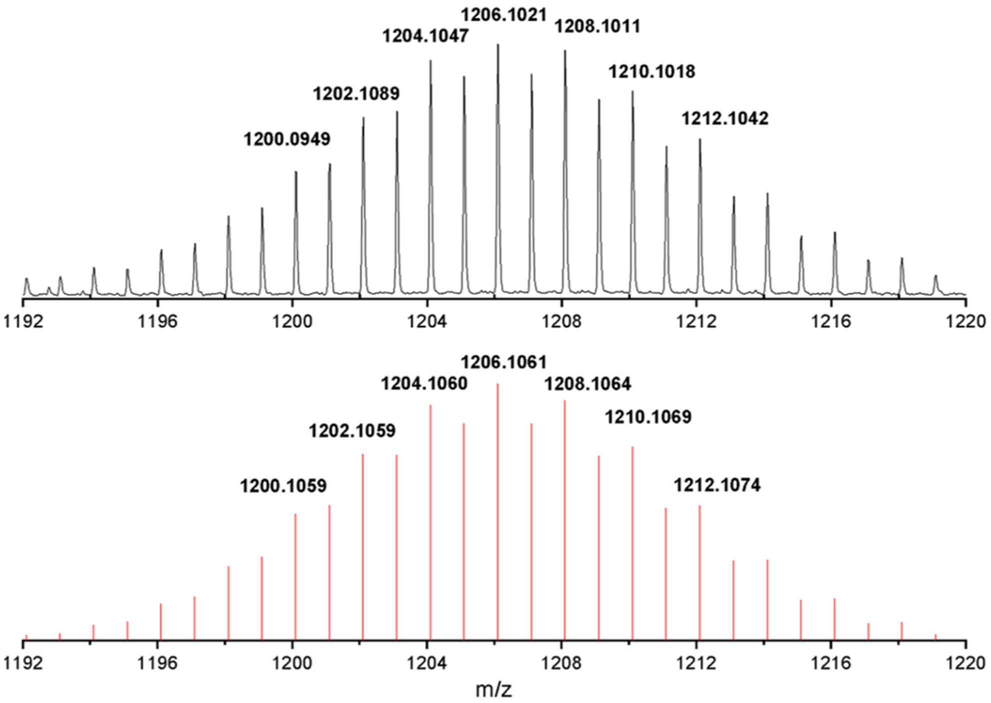

Supplementary Figure 23. Measured (top) and simulated (bottom) spectrum of the fragment $[Mo_2(CO)_6Sn_2Sb_5]^-$.

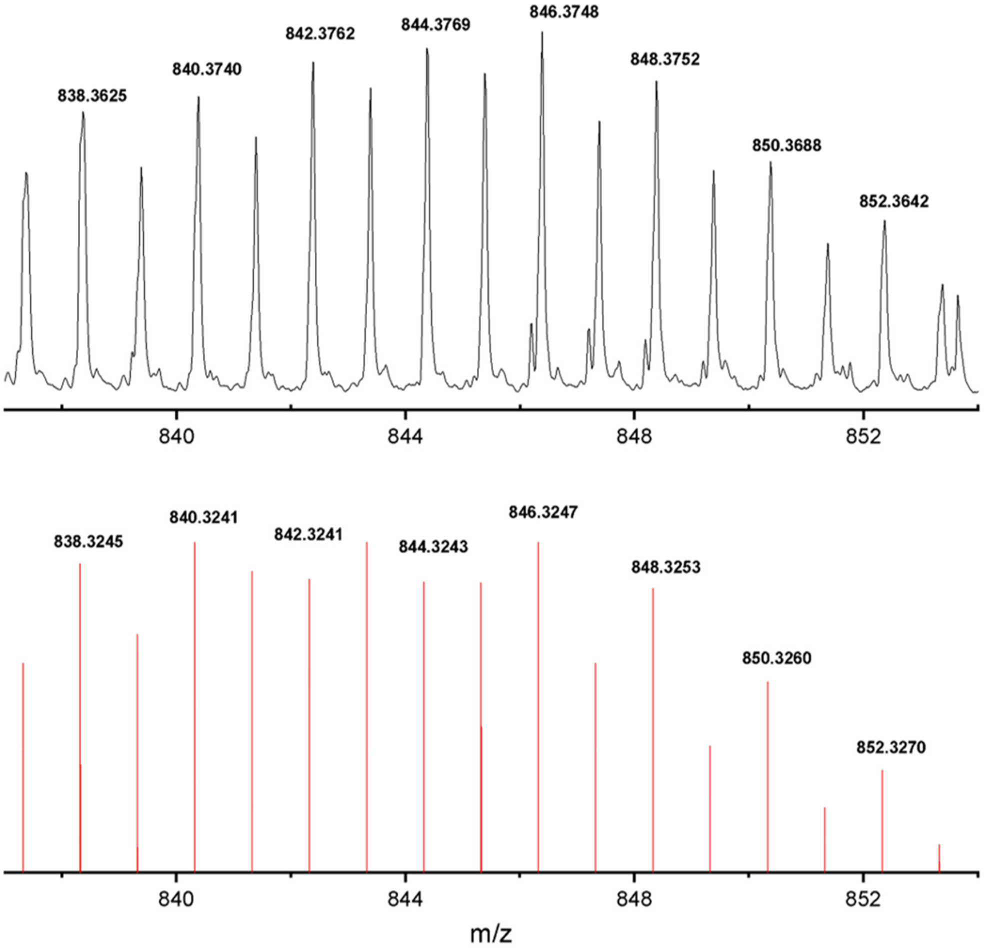

Supplementary Figure 24. Measured (top) and simulated (bottom) spectrum of the combined fragments $[Sn_2Sb_5]^-$, $[Sn_3Sb_4]^-$ and $[Sn_4Sb_3]^-$.

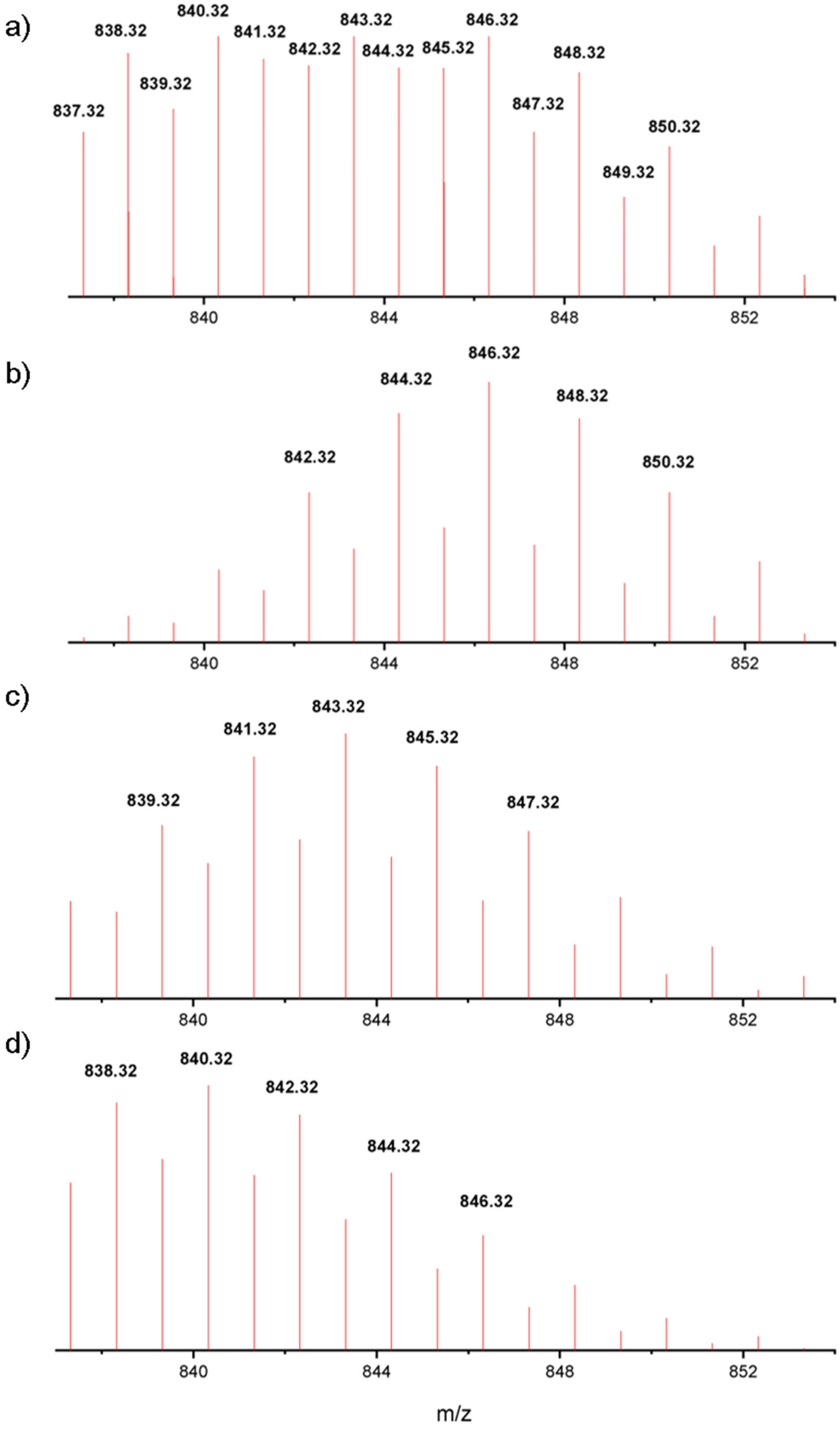

Supplementary Figure 25. Simulated spectrum of the fragments, a) overview, b) $[Sn_2Sb_5]^-$, c) $[Sn_3Sb_4]^-$, d) $[Sn_4Sb_3]^-$ (The relative contribution is 1:1:1, respectively).

3.Energy Dispersive X-ray (EDX) Spectroscopic Analysis

The results of EDX analysis on **1'**, **2'**, **3'**, and **4'** are presented in Supplementary Figures 26-29 respectively. A deviation of the amount of K is rather common to be observed in the EDX characterization of Zintl clusters which can be ascribed to the irregular surfaces of the crystals after exposure to air. A relatively small deviation of Sn content is observed in compound **3'** which can be caused by the adhesion of other kinds of metal powder to the crystal surface.

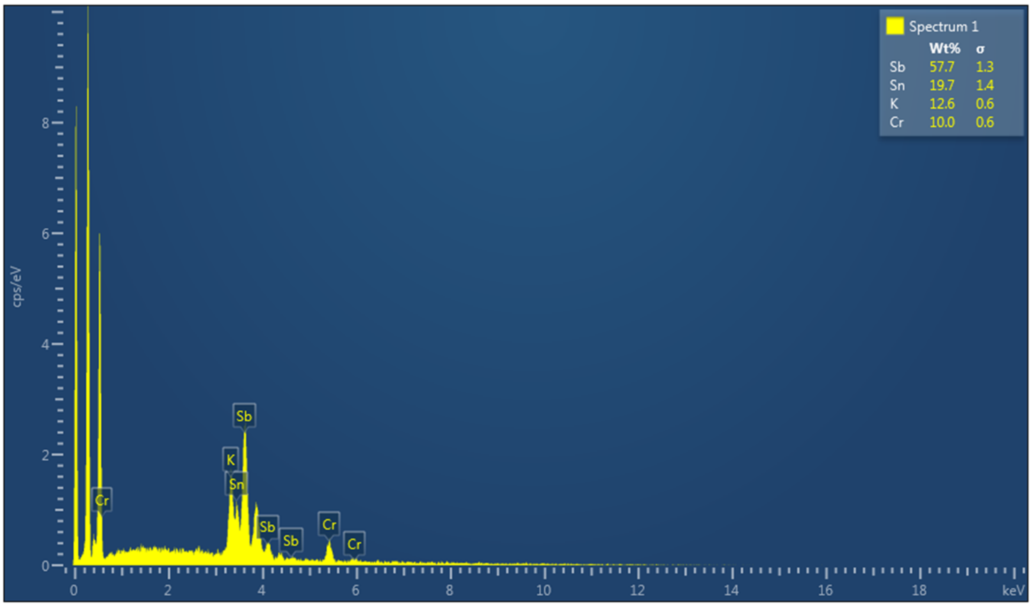

Element	Line type	wt%	σ	Experimental / Calculated Atom %
K	K series	12.6	0.6	27.91/25.00
Cr	L series	10.0	0.6	16.65/16.67
Sn	L series	19.7	1.4	14.38/16.67
Sb	L series	57.7	1.3	41.05/41.67

Supplementary Figure 26. EDX analysis of compound **1'**.

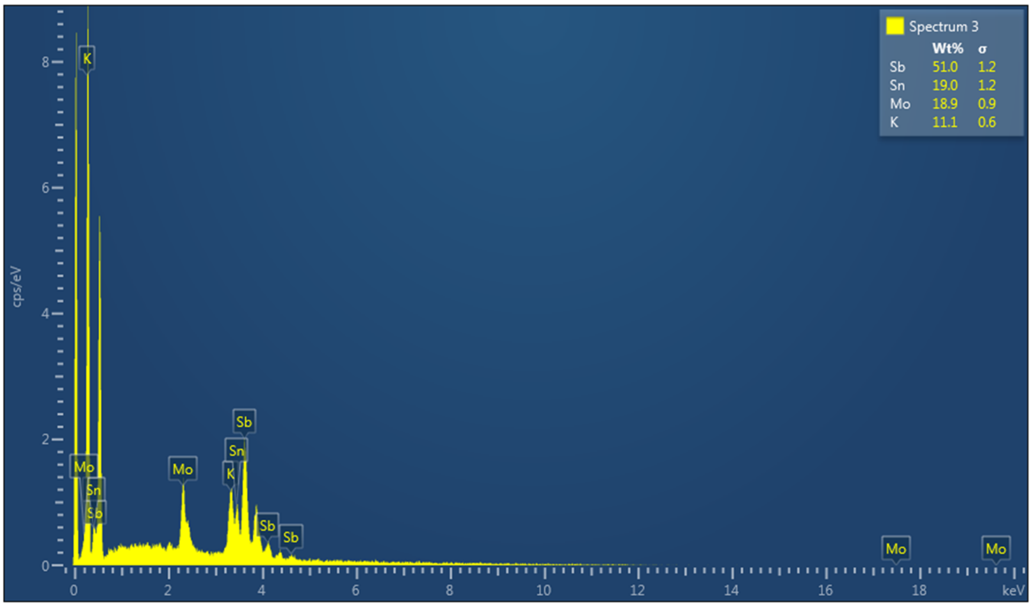

Element	Line type	wt%	σ	Experimental / Calculated Atom %
K	K series	11.1	0.6	26.78/25.00
Mo	L series	18.9	0.9	18.58/16.67
Sn	L series	19.0	1.2	15.10/16.67
Sb	L series	51.0	1.2	39.53/41.67

Supplementary Figure 27. EDX analysis of compound **2’**.

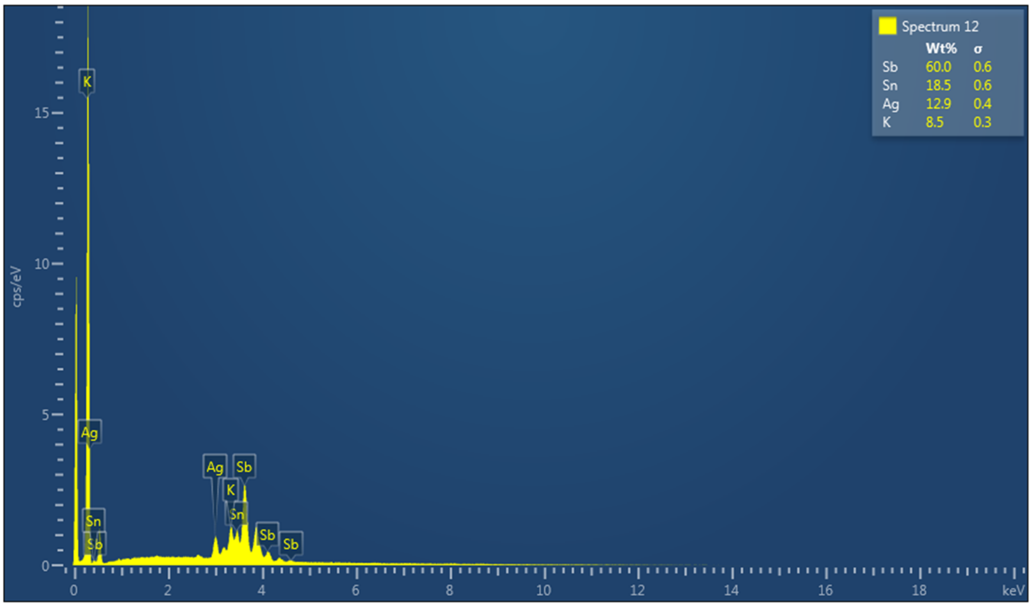

Element	Line type	wt%	σ	Experimental / Calculated Atom %
K	K series	8.5	0.3	22.06/20.00
Ag	L series	12.9	0.4	12.14/10.00
Sn	L series	18.5	0.6	15.82/20.00
Sb	L series	60.0	0.6	49.98/50.00

Supplementary Figure 28. EDX analysis of compound **3'**.

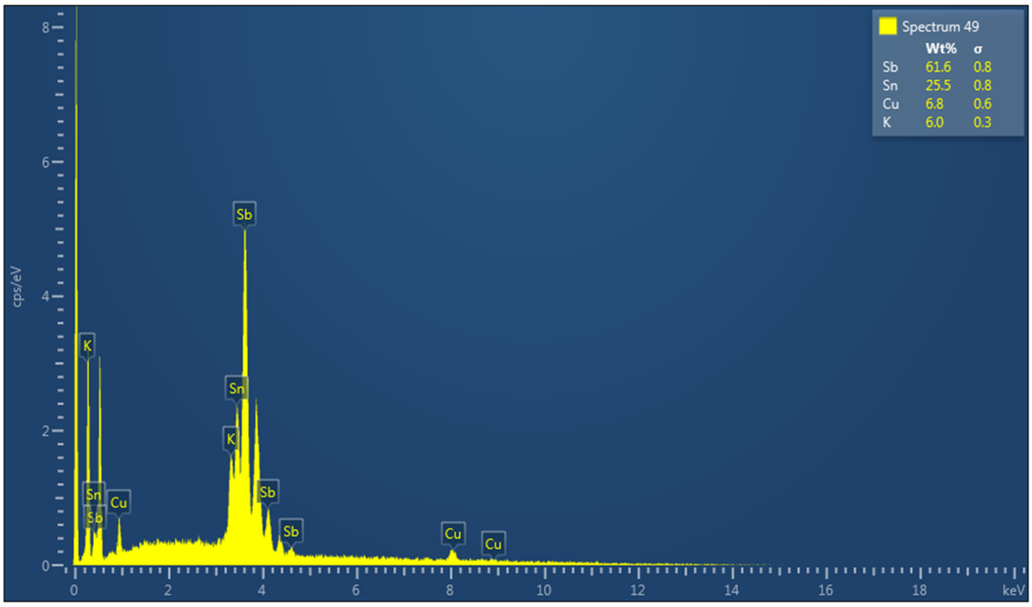

Element	Line type	wt%	σ	Experimental / Calculated Atom %
K	K series	6.0	0.3	15.65/20.00
Cu	L series	6.8	0.6	10.84/10.00
Sn	L series	25.5	0.8	21.91/20.00
Sb	L series	61.6	0.8	53.61/50.00

Supplementary Figure 29. EDX analysis of compound **4'**.

4. Supplementary Computational Data.

Discussion of chemical bonding of $M(CO)_3$ fragments for clusters 1 and 2:

The chemical bonding of the central part of $[Mo_2(CO)_6Sn_2Sb_5]^{3-}$ and $[Cr_2(CO)_6Sn_2Sb_5]^{3-}$ are discussed in the main text and covers 18 two-electron bonding elements. The remaining 88 valence electrons are responsible for the bonding between C and O atoms in the CO units and their coordination to the transition metal atoms. Both clusters possess similar chemical bonding and for clarity, further discussion will be about Mo-containing structure. The complete bonding pattern of the Mo-CO fragment is shown in Supplementary Figure 32 There are six s-type lone pairs on O-atoms, six 2c-2e C-O σ-bonds, and twelve 2c-2e C-O π

-bonds accounting for the triple C≡O bonds. Coordination of the six CO molecules to the two Mo-atoms is manifested through six 2c-2e C-Mo σ-bonds with ON = 1.98 |e| and six 3c-2e π bonds with ON = 1.73-1.72 |e| that are responsible for the π-back-donation from *d*-type atomic orbital of Mo to the π* orbital of the CO ligand. The contribution of the *d*-lone pairs of Mo atoms to the 3c-2e fragments is ~85%. The presence of the π backdonation is evident by the somewhat longer C-O bond lengths, which are increased by ~0.05 Å as compared to the optimized geometry of the CO molecule at the same level of theory (Supplementary Table 9).

Supplementary Table 6. The lowest metastable isomers and their relative energies of $[Sn_2Sb_5]^{3-}$ units as found by the CK algorithm at different levels of theory.

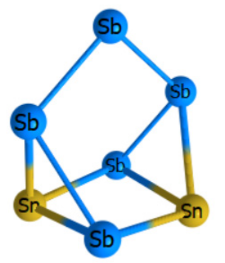	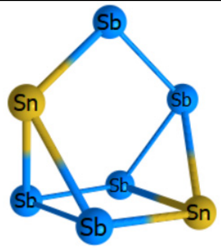	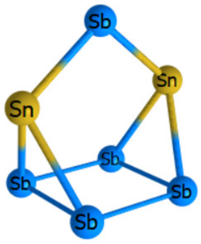	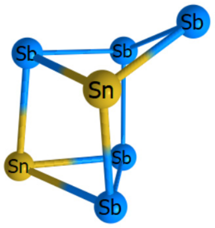
C2-$[Sn_2Sb_5]^{3-}$	*C1*-$[Sn_2Sb_5]^{3-}$	*C2v*-$[Sn_2Sb_5]^{3-}$	*C1*-$[Sn_2Sb_5]^{3-}$
PBE0/def2-TZVP: 0.0 kcal/mol<br>BP86/def2-TZVP: 0.0 kcal/mol<br>B3LYP/def2-TZVP: 0.0 kcal/mol<br>M06/def2-TZVP: 0.0 kcal/mol<br>M06-2X/def2-TZVP: 0.0kcal/mol	PBE0/def2-TZVP: 1.6 kcal/mol<br>BP86/def2-TZVP: 1.3 kcal/mol<br>B3LYP/def2-TZVP: 1.4 kcal/mol<br>M06/def2-TZVP: 1.3 kcal/mol<br>M06-2X/def2-TZVP: 2.6 kcal/mol	PBE0/def2-TZVP: 3.5 kcal/mol<br>BP86/def2-TZVP: 2.8 kcal/mol<br>B3LYP/def2-TZVP: 3.2 kcal/mol<br>M06/def2-TZVP: 3.1 kcal/mol<br>M06-2X/def2-TZVP: 5.0 kcal/mol	PBE0/def2-TZVP: 6.0 kcal/mol<br>BP86/def2-TZVP: 6.2 kcal/mol<br>B3LYP/def2-TZVP: 6.6 kcal/mol<br>M06/def2-TZVP: 4.7 kcal/mol<br>M06-2X/def2-TZVP: 6.7 kcal/mol
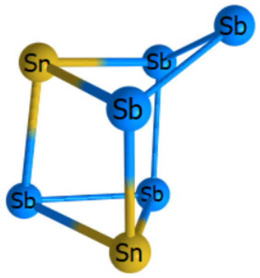	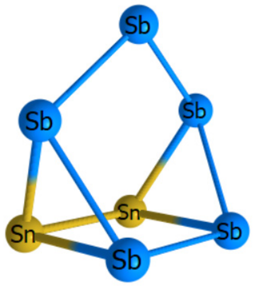	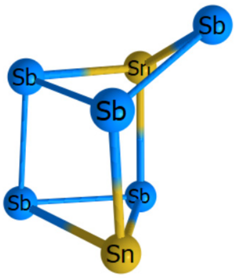	
C1-$[Sn_2Sb_5]^{3-}$	*Cs*-$[Sn_2Sb_5]^{3-}$	*C1*-$[Sn_2Sb_5]^{3-}$	
PBE0/def2-TZVP: 6.3 kcal/mol<br>BP86/def2-TZVP: 6.5 kcal/mol<br>B3LYP/def2-TZVP: 6.6 kcal/mol<br>M06/def2-TZVP: 4.7 kcal/mol<br>M06-2X/def2-TZVP: 6.9kcal/mol	PBE0/def2-TZVP: 7.0 kcal/mol<br>BP86/def2-TZVP: 6.3 kcal/mol<br>B3LYP/def2-TZVP: 6.8 kcal/mol<br>M06/def2-TZVP: 6.0 kcal/mol<br>M06-2X/def2-TZVP: 9.7 kcal/mol	PBE0/def2-TZVP: 8.1 kcal/mol<br>BP86/def2-TZVP: 7.9 kcal/mol<br>B3LYP/def2-TZVP: 8.2 kcal/mol<br>M06/def2-TZVP: 6.1 kcal/mol<br>M06-2X/def2-TZVP: 9.0 kcal/mol	

Supplementary Table 7. The lowest energy isomers and their relative energies of $K_3[Sn_2Sb_5]$ as found by CK algorithm at different levels of theory.

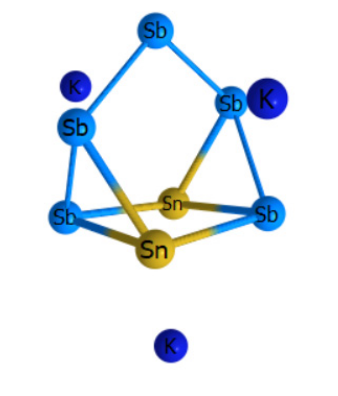	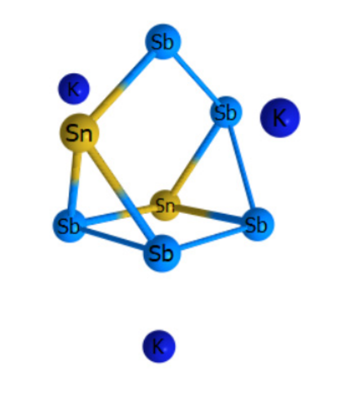	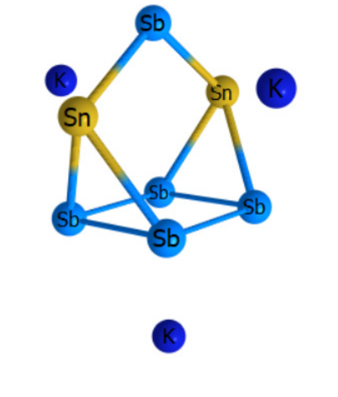	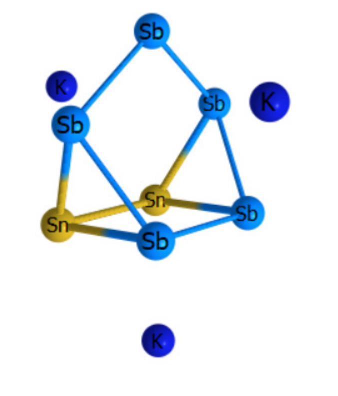
C1-$K_3[Sn_2Sb_5]$	*C1*- $K_3[Sn_2Sb_5]$	*C2v*- $K_3[Sn_2Sb_5]$	*Cs*- $K_3[Sn_2Sb_5]$
PBE0/def2-TZVP: 0.00 kcal/mol<br>BP86/def2-TZVP: 0.00 kcal/mol<br>B3LYP/def2-TZVP: 0.04 kcal/mol<br>M06/def2-TZVP: 0.00 kcal/mol<br>M06-2X/def2-TZVP: 0.00 kcal/mol<br>CCSD/ def2-TZVP//PBE0/def2-TZVP: 0.00 kcal/mol	PBE0/def2-TZVP: 0.40 kcal/mol<br>BP86/def2-TZVP: 0.09 kcal/mol<br>B3LYP/def2-TZVP: 0.00 kcal/mol<br>M06/def2-TZVP: 0.14 kcal/mol<br>M06-2X/def2-TZVP: 0.12 kcal/mol<br>CCSD/ def2-TZVP//PBE0/def2-TZVP: 0.57 kcal/mol	PBE0/def2-TZVP: 1.38 kcal/mol<br>BP86/def2-TZVP: 0.77 kcal/mol<br>B3LYP/def2-TZVP: 0.77 kcal/mol<br>M06/def2-TZVP: 1.09 kcal/mol<br>M06-2X/def2-TZVP: 1.03 kcal/mol<br>CCSD/ def2-TZVP//PBE0/def2-TZVP: 1.70 kcal/mol	PBE0/def2-TZVP: 8.10 kcal/mol<br>BP86/def2-TZVP: 7.35 kcal/mol<br>B3LYP/def2-TZVP: 7.93 kcal/mol<br>M06/def2-TZVP: 7.04 kcal/mol<br>M06-2X/def2-TZVP: 9.88 kcal/mol<br>CCSD/ def2-TZVP//PBE0/def2-TZVP: 8.66 kcal/mol
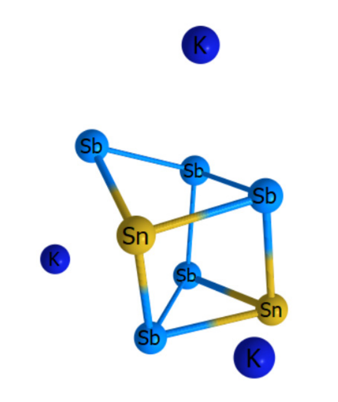	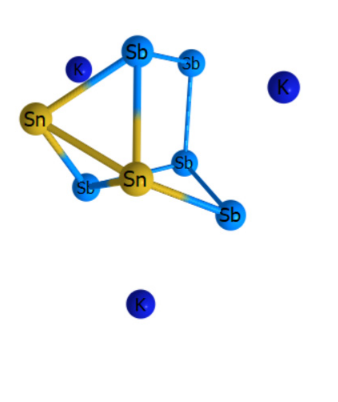	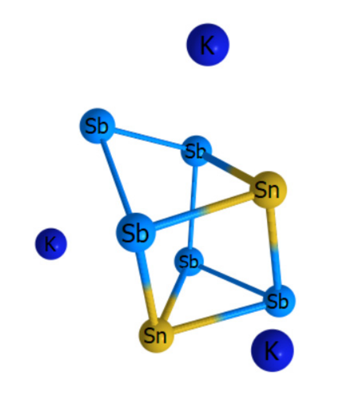	
C1- $K_3[Sn_2Sb_5]$	*C1*- $K_3[Sn_2Sb_5]$	*C1*- $K_3[Sn_2Sb_5]$	
PBE0/def2-TZVP: 11.46 kcal/mol<br>BP86/def2-TZVP: 11.36 kcal/mol<br>B3LYP/def2-TZVP: 11.04 kcal/mol<br>M06/def2-TZVP: 10.20 kcal/mol<br>M06-2X/def2-TZVP: 10.43 kcal/mol	PBE0/def2-TZVP: 14.83 kcal/mol<br>BP86/def2-TZVP: 12.78 kcal/mol<br>B3LYP/def2-TZVP: 12.52 kcal/mol<br>M06/def2-TZVP: 12.60 kcal/mol<br>M06-2X/def2-TZVP: 13.99 kcal/mol	PBE0/def2-TZVP: 18.08 kcal/mol<br>BP86/def2-TZVP: 17.70 kcal/mol<br>B3LYP/def2-TZVP: 17.69 kcal/mol<br>M06/def2-TZVP: 15.79 kcal/mol<br>M06-2X/def2-TZVP: 19.06 kcal/mol	

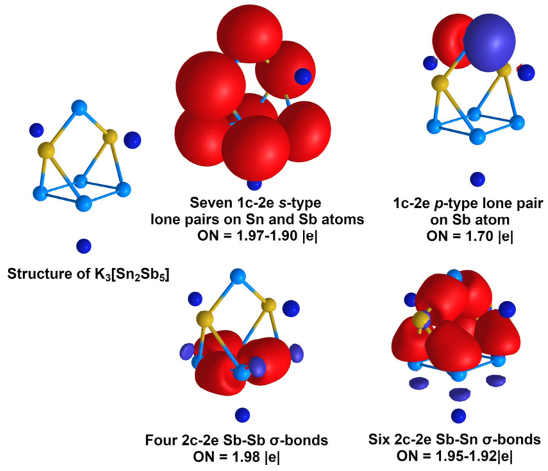

Supplementary Figure 30. Chemical bonding pattern of *C2v*-$K_3[Sn_2Sb_5]$. ON denotes occupation numbers, here and elsewhere (equal to 2.00 |e| in an ideal case). Lines between atoms are presented for visualization and do not necessarily correspond to 2c-2e bonds, here and elsewhere. Sn-atoms are dark yellow, Sb-atoms are blue, K-atoms are dark-blue.

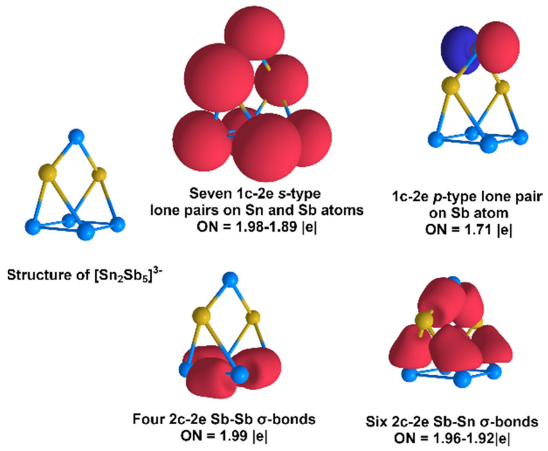

Supplementary Figure 31. Chemical bonding pattern of $[Sn_2Sb_5]^{3-}$. Sn-atoms are dark yellow , Sb-atoms are blue.

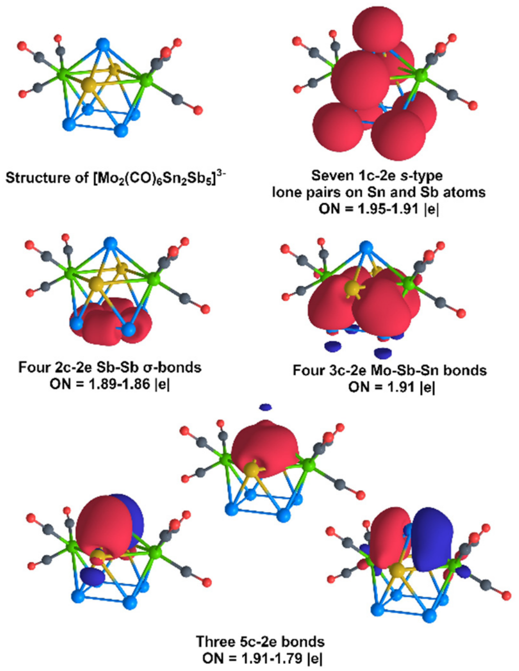

Supplementary Figure 32. Chemical bonding pattern of a cage fragment of $[Mo_2(CO)_6Sn_2Sb_5]^{3-}$. Sn-atoms are dark yellow, Sb-atoms are blue, Mo-atoms are green, C-atoms are grey, and O-atoms are red.

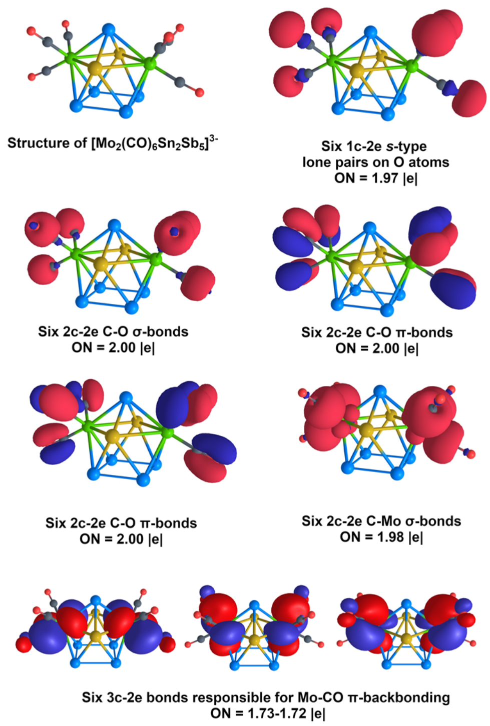

Supplementary Figure 33. Chemical bonding pattern of Mo-CO fragments of $[Mo_2(CO)_6Sn_2Sb_5]^{3-}$. Sn-atoms are dark yellow, Sb-atoms are blue, Mo-atoms are green, C-atoms are grey, and O-atoms are red.

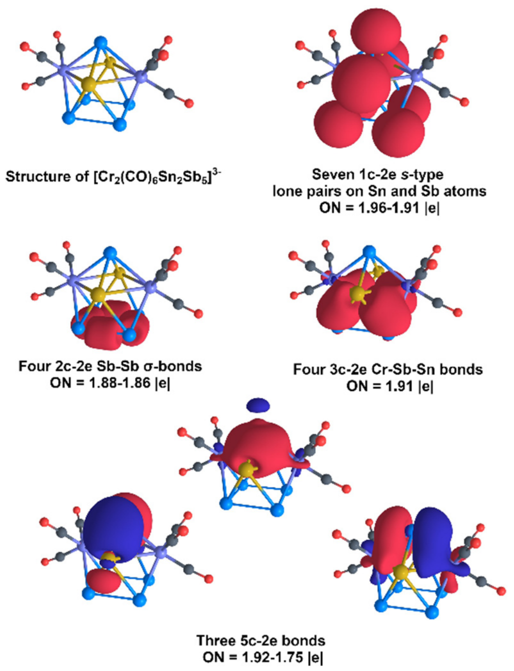

Supplementary Figure 34. Chemical bonding pattern of a cage fragment of $[Cr_2(CO)_6Sn_2Sb_5]^{3-}$. Sn-atoms are dark yellow, Sb-atoms are blue, Cr-atoms are purple, C-atoms are grey, and O-atoms are red.

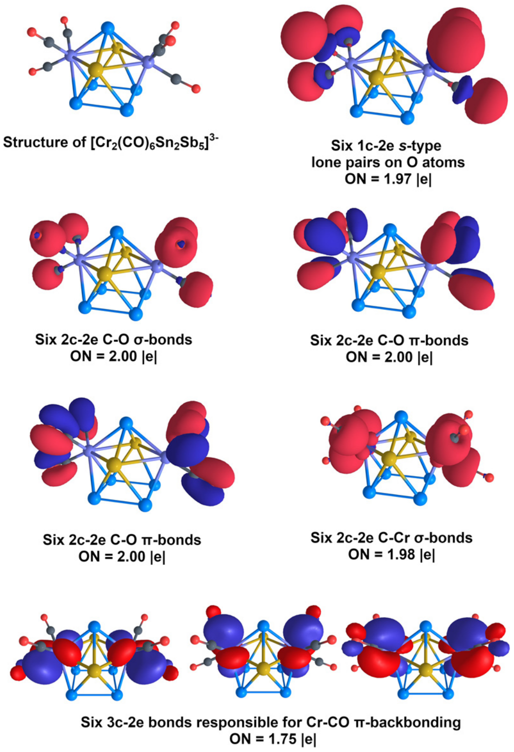

Supplementary Figure 35. Chemical bonding pattern of Cr-CO fragment of $[Cr_2(CO)_6Sn_2Sb_5]^{3-}$. Sn-atoms are dark yellow, Sb-atoms are blue, Cr-atoms are purple, C-atoms are grey, and O-atoms are red.

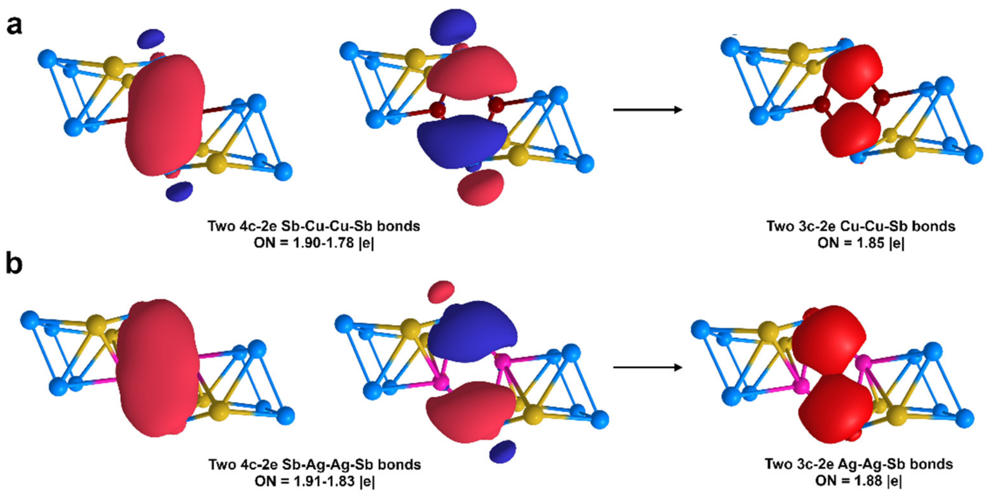

Supplementary Figure 36. Chemical bonding pattern of M_2Sb_2 fragments (M = Cu (a); Ag (b)). Sn-atoms are dark yellow, Sb-atoms are blue, Ag-atoms are pink, Cu-atoms are dark red.

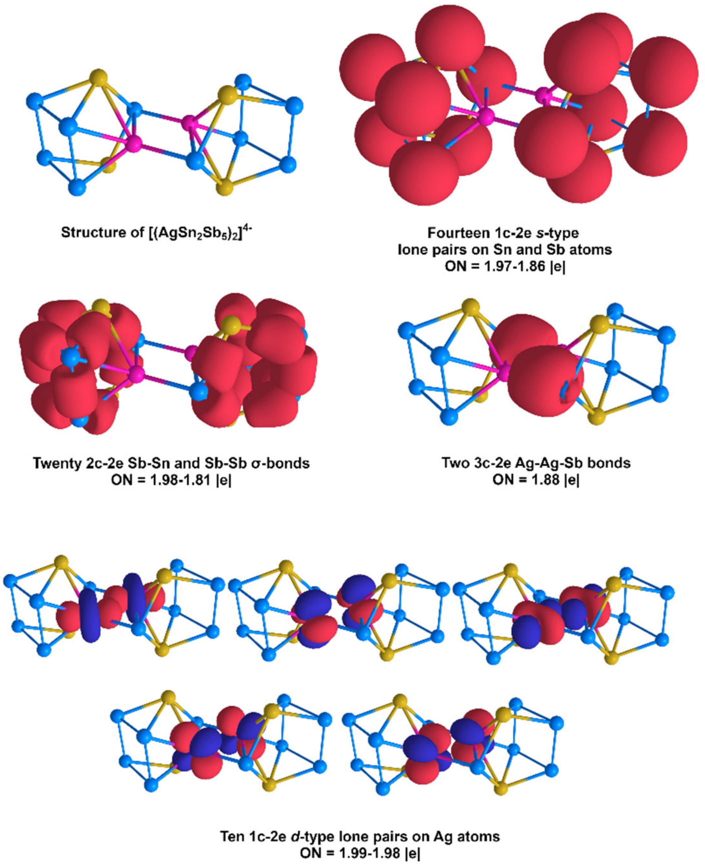

Supplementary Figure 37. Chemical bonding pattern of $[(AgSn_2Sb_5)_2]^{4-}$. Sn-atoms are dark yellow, Sb-atoms are blue, Ag-atoms are pink.

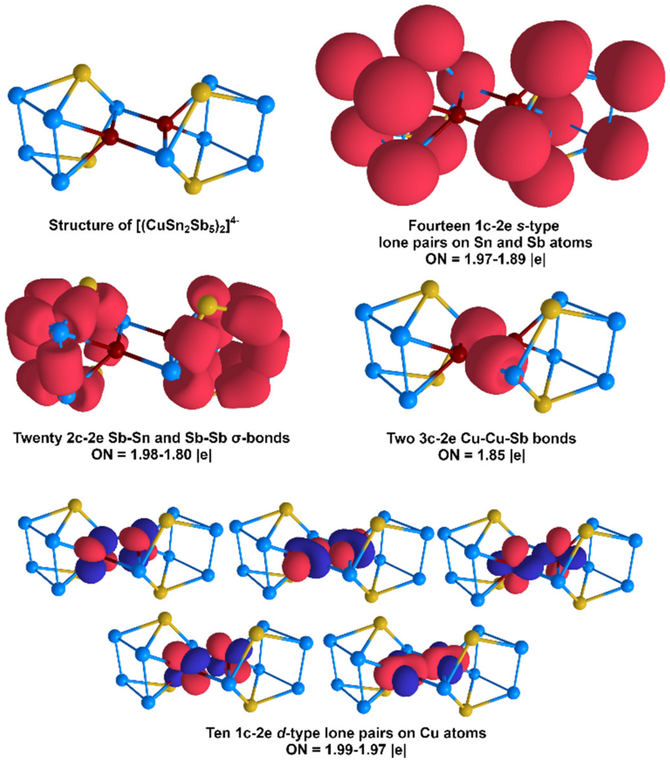

Supplementary Figure 38. Chemical bonding pattern of $[(CuSn_2Sb_5)_2]^{4-}$. Sn-atoms are dark yellow, Sb-atoms are blue, Cu-atoms are dark red.

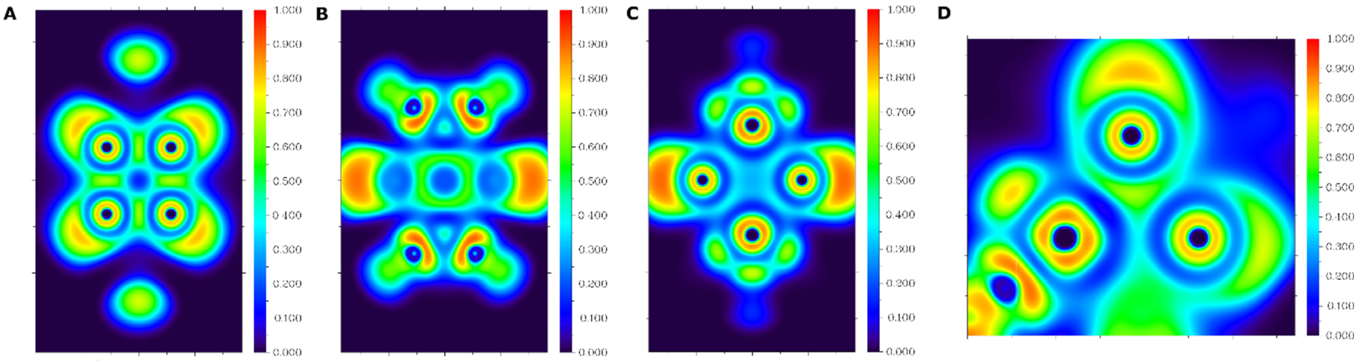

Supplementary Figure 39. The 2D representation of ELF for selected fragments of $[Mo_2(CO)_6Sn_2Sb_5]^{3-}$ cluster. a) the Sb_4 square fragment; b) the plane illustrating the middle of the $SbMo_2Sn_2$ cap parallel to Mo_2Sn_2 base; c) the Mo_2Sn_2 base of the $SbMo_2Sn_2$ cap; d) Mo-Sn-Sb triangle fragment (from left to right: Mo-atom, Sb-atom, Sn-atom)

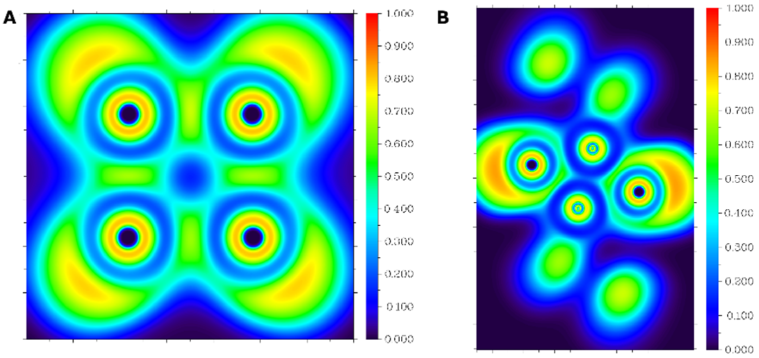

Supplementary Figure 40. The 2D representation of ELF for selected fragments of $[(CuSn_2Sb_5)_2]^{4-}$ cluster. a) the Sb_4 square fragment; b) the Cu_2Sb_2 diamond fragment.

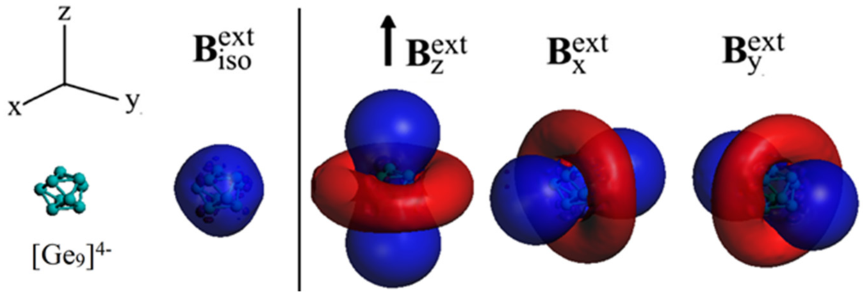

Supplementary Figure 41. Plots of magnetic response of $[Ge_9]^{4-}$. The induced magnetic field for involving an orientation averaged (B_{iso}^{ind} and to the different orientation of the external field along three representative axes for $[Ge_9]^{4-}$. Isovalue set to ±2 ppm. Blue represent shielding surfaces and red, deshielding surfaces.

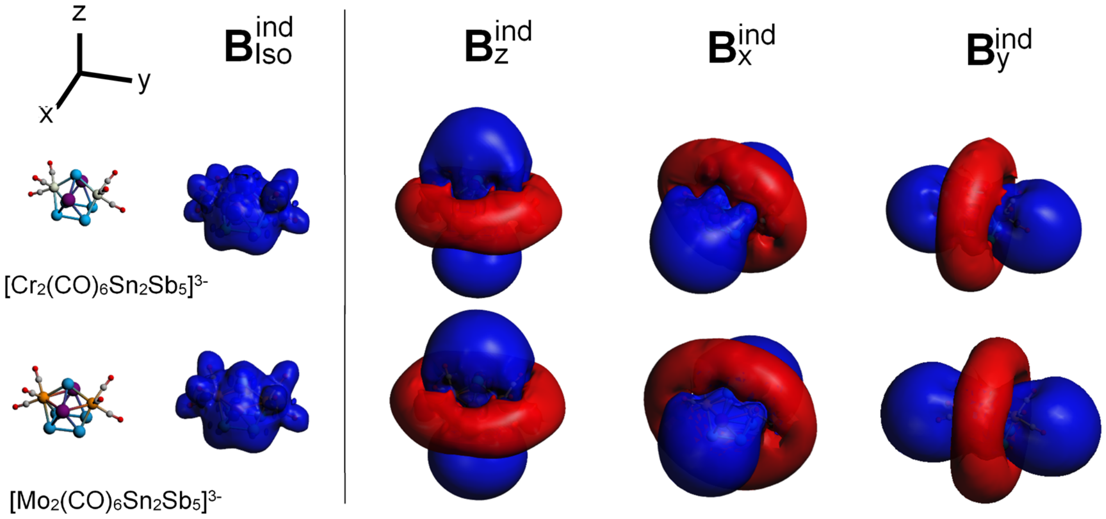

Supplementary Figure 42. Plots of magnetic response of the studied clusters 1 and 2. Induced magnetic field accounting for the orientation averaged (B^{ind}_{iso}) term, and for different orientation of the external field along three

representative axes, for $[Cr_2(CO)_6Sn_2Sb_5]^{3-}$ and $[Mo_2(CO)_6Sn_2Sb_5]^{3-}$. Blue surface: Shielding; Red surface: Deshielding. Isovalue set to ± 2 ppm.

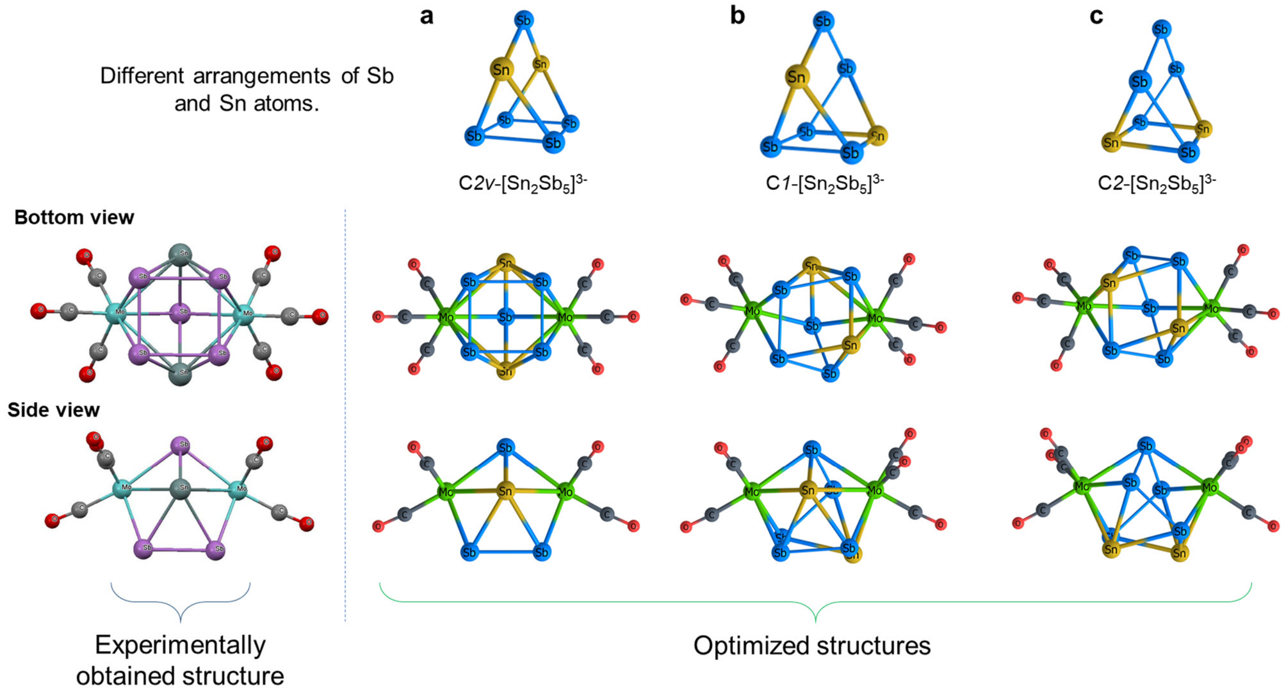

Supplementary Figure 43. The comparison of experimentally obtained structure of $[Mo_2(CO)_6Sn_2Sb_5]^{3-}$ and optimized structures with different positions of the Sb and Sn atoms in the $[Sn_2Sb_5]^{3-}$ units.

Supplementary Table 8. $NICS_{iso}$ and $NICS_{zz}$ indices calculated for $\{[AgSn_2Sb_5]_2\}^{4-}$ and $\{[CuGe_9Mes]_2\}^{4-}$.

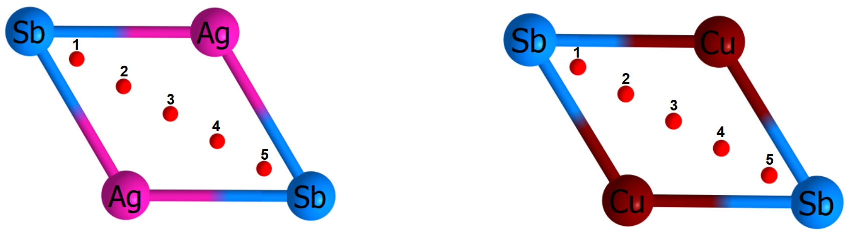

	$\{[AgSn_2Sb_5]_2\}^{4-}$		$\{[CuGe_9Mes]_2\}^{4-}$	
Point	$NICS_{iso}$	$NICS_{zz}$	$NICS_{iso}$	$NICS_{zz}$
1	-37.88	-6.26	-46.92	-12.59
2	-22.41	8.55	-27.42	-1.30
3	-24.91	11.08	-28.16	2.23
4	-22.41	8.55	-27.42	-1.30
5	-37.88	-6.26	-46.92	-12.59
