# Supplementary material for: Ternary aromatic and anti-aromatic clusters derived from the hypho species [Sn2Sb5]3−
Source: Nat Commun. 2021 Jul 22;12:4465. doi: 10.1038/s41467-021-24706-4 (PMC8298489; doi:10.1038/s41467-021-24706-4)
Supplement: Supplementary file 3 — Description of Additional Supplementary Files [file 41467_2021_24706_MOESM3_ESM.pdf]

Description of Additional Supplementary Files

Supplementary Data 1: Cartesian coordinates of investigated clusters (structures were optimized at PBE0/def2-TZVP level of theory).
